# Draft Genome of White-blotched River Stingray Provides Novel Clues for Niche Adaptation and Skeleton Formation

**DOI:** 10.1016/j.gpb.2022.11.005

**Published:** 2022-12-05

**Authors:** Jingqi Zhou, Ake Liu, Funan He, Yunbin Zhang, Libing Shen, Jun Yu, Xiang Zhang

**Affiliations:** 1 School of Public Health, Shanghai Jiao Tong University School of Medicine, Shanghai 200025, China; 2 Department of Life Sciences, Changzhi University, Changzhi 046011, China; 3 Greehey Children’s Cancer Research Institute, UT Health San Antonio, San Antonio, TX 78229, USA; 4 State Key Laboratory of Cell Biology, Shanghai Key Laboratory of Molecular Andrology, Shanghai Institute of Biochemistry and Cell Biology, Center for Excellence in Molecular Cell Science, Chinese Academy of Sciences, Shanghai 200031, China; 5 International Human Phenome Institutes (Shanghai), Shanghai 200433, China; 6 Institute of Neuroscience, Center for Excellence in Brain Science and Intelligence Technology, Chinese Academy of Sciences, Shanghai 200031, China; 7 Beijing Institute of Genomics, Chinese Academy of Sciences and China National Center for Bioinformation, Beijing 100101, China; 8 University of Chinese Academy of Sciences, Beijing 100049, China; 9 Shanghai Nanmulin Biotechnology Company Limited, Shanghai 200031, China

**Keywords:** White-blotched river stingray, *Potamotrygon leopoldi*, *De novo* genome assembly, Vitamin D-binding protein, Niche adaptation

## Abstract

The **white-blotched river stingray** (***Potamotrygon leopoldi***) is a cartilaginous fish native to the Xingu River, a tributary of the Amazon River system. As a rare freshwater-dwelling cartilaginous fish in the Potamotrygonidae family in which no member has the genome sequencing information available, *P*. *leopoldi* provides the evolutionary details in fish phylogeny, **niche adaptation**, and skeleton formation. In this study, we present its draft genome of 4.11 Gb comprising 16,227 contigs and 13,238 scaffolds, with contig N50 of 3937 kb and scaffold N50 of 5675 kb in size. Our analysis shows that *P*. *leopoldi* is a slow-evolving fish that diverged from elephant sharks about 96 million years ago. Moreover, two gene families related to the immune system (immunoglobulin heavy constant delta genes and T-cell receptor alpha/delta variable genes) exhibit expansion in *P*. *leopoldi* only. We also identified the Hox gene clusters in *P*. *leopoldi* and discovered that seven *Hox* genes shared by five representative fish species are missing in *P*. *leopoldi*. The RNA sequencing data from *P*. *leopoldi* and other three fish species demonstrate that fishes have a more diversified tissue expression spectrum when compared to mammals. Our functional studies suggest that lack of the *gc* gene encoding **vitamin D-binding protein** in cartilaginous fishes (both *P*. *leopoldi* and *Callorhinchus milii*) could partly explain the absence of hard bone in their endoskeleton. Overall, this genome resource provides new insights into the niche adaptation, body plan, and skeleton formation of *P. leopoldi*, as well as the genome evolution in cartilaginous fishes.

## Introduction

The transition of jawless to jawed vertebrates lays a foundation for the evolution of vertebrates, which was accompanied by many morphological and phenotypic innovations, especially jaws and the adaptive immune system [1]. As ancient jawed vertebrates, the fish constitutes a highly diverse and evolutionarily successful class found in both marine and freshwater habitats [2]. The jawed fishes belong to two clades, the cartilaginous fishes (Chondrichthyes) and bony vertebrates (Osteichthyes), which diverged about 450 million years ago (MYA) [3]. Cartilaginous fishes are the most basal group of living fishes, which contain about 1000 living species [4]. Except for a few published genome information of Chondrichthyes [5], [6], [7], too few genetic data in this important taxonomic position are available for scientists to further study the evolution of chordates and the origin of hard bone formation.

The white-blotched river stingray (*Potamotrygon leopoldi*), also known as Xingu River ray, is a freshwater cartilaginous fish native to the Xingu River basin in Brazil [8]. The Xingu River is a geographical part of the Amazon River basin, which was inundated by sea during the Pleistocene Epoch. The ancestor of this stingray experienced the transition from marine to freshwater environment. *P*. *leopoldi* belongs to the family Potamotrygonidae in the order Myliobatiformes composed of a group of cartilaginous fishes most-closely related to sharks [9]. The species under the family Potamotrygonidae all live in the tropical and subtropical regions of South America [9]. Unlike the freshwater stingrays in Africa, Asia, and Australia, which belong to the family Dasyatidae, most Potamotrygonidae species live strictly in freshwater, whereas most Dasyatidae species are saltwater dwellers [10], [11]. Except a few widespread members, most river stingrays typically reside in and are confined to a single river basin [10]. For its unique appearance (*e.g.*, white spots on black skin) and distinct behavior (*e.g.*, swimming-maneuvering capabilities), *P*. *leopoldi* becomes a pricy pet fish popular in home- and office-based aquaria. Till now, no fish species from the family Potamotrygonidae has been extensively studied at the genome level. Whole-genome data of a Potamotrygonidae member and its comparative analysis with other available fish genomes might help us further reveal the evolutionary features unique to Potamotrygonidae and provide insights into the ancestral state of gnathostome-specific morphological characters and physiological systems. Therefore, *P. leopoldi* provides an excellent model for studying evolution and niche adaptation of freshwater cartilaginous fishes.

In this study, we assembled a 4.11-Gb genome of a male stingray, *P*. *leopoldi*, using the whole-genome shotgun (WGS) approach and based on a raw data collection with a total of 370.97× genome coverage, generated from Pacific Biosciences (PacBio) single molecule real time sequencing (SMRT), Illumina HiSeq2000, and 10X Genomics sequencing platforms. We subsequently compared its genome to other five representative fish genomes (*Cyprinus carpio*, *Lepisosteus oculatus*, *Latimeria chalumnae*, *Danio rerio*, and *Callorhinchus milii*) and one chordate genome (*Branchiostoma floridae*), to capture its unique evolutionary features and molecular basis. Our results indicate that *P*. *leopoldi* is one of the slowest-evolving fish species, even within the cartilaginous fish lineage. The transcriptomic data, obtained from six tissues of *P*. *leopoldi*, shed further lights into highly diversified gene expression profiles among fish lineages, as opposed to the highly coordinated gene expression among mammalian tissues. The knockdown experiment in the fish model reveals the possible genetic foundation for the divergence of hard and cartilaginous skeleton formations. Together, our results start from the *P*. *leopoldi* genome sequencing to the experimental model, providing novel clues for niche adaptation and skeleton formation in the evolutionary history of fish.

## Results

### *P*. *leopoldi* with a draft genome assembly of 4.11 Gb

We constructed a total of 17 sequencing libraries using genomic DNA extracted from a male *P*. *leopoldi*, and acquired raw data from three sequencing platforms, PacBio SMRT, Illumina HiSeq2000, and 10X Genomics, with coverages of 61.61×, 200.95×, and 108.41×, respectively (Table S1). After stringent filtering and redundancy checking, 1628.63-Gb sequence data were used for a scaffold-based *de novo *genome assembly. The initial combined assembly was based on the data from PacBio long reads. Illumina paired-end data and 10X Genomics data were used for error correction. The DNA composition of the assembled contigs was of 41.97% GC content (Table S2). The genome size estimated by *K*-mer analysis using Illumina paired-end data was about 4.11 Gb in size with 0.79% heterozygosity (Table S3). The read-to-genome alignment rate is 98.48% with a coverage of 98.74% in the assembly (Table S4). The final assembly consisted of 16,227 contigs and 13,238 scaffolds, with a contig N50 of 3937 kb and a scaffold N50 of 5675 kb in size (Table S5). The completeness of the *P*. *leopoldi* genome was estimated to achieve 91.8% (3081/3354) coverage using the Benchmarking Universal Single-Copy Orthologs (BUSCO) method. We mapped 248 core eukaryotic genes (CEGs) to the scaffolds, and 90% of them are found in the predicted exons, based on BLAST-like alignment tool (BLAT) scores. Additionally, Merqury gave the accuracy in consensus base calling with 99.9% (Q30) for the genome assembly (Table 1). Taken together, the BUSCO results, CEG results, and mapping quality indicate that our genome assembly is highly accurate and nearly complete.

**Table 1:** **Merqury metrics for drafts of the *P*. *leopoldi* genome**

**Metric**	**Genome**
Consensus quality value	37.53
Assembly error rate (%)	< 0.01
*K*-mer completeness (%)	96.51

### More than 90% of ***P***. ***leopoldi*** genes have known functions

**Supplementary Figure S1:**
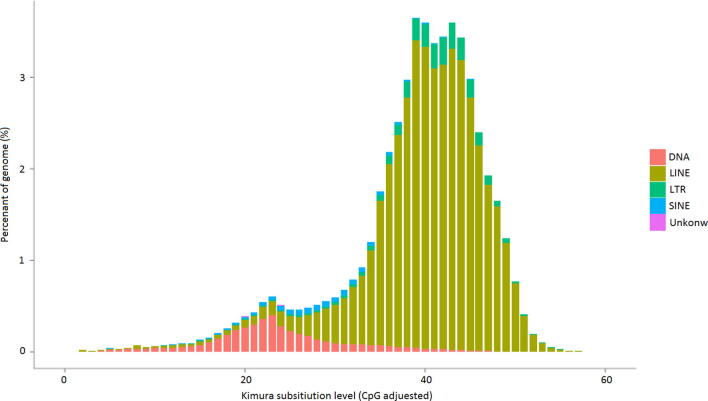
**The distribution of tandem repeat sequences in white-blotched river stingray genome** X-axis is the divergence of stingray’s tandem repeat sequences to the TE sequences in the Repbase database. SINE, short interspersed element; LINE, long interspersed element; LTR, long terminal repeat; TE, transposon element.

**Supplementary Figure S2:**
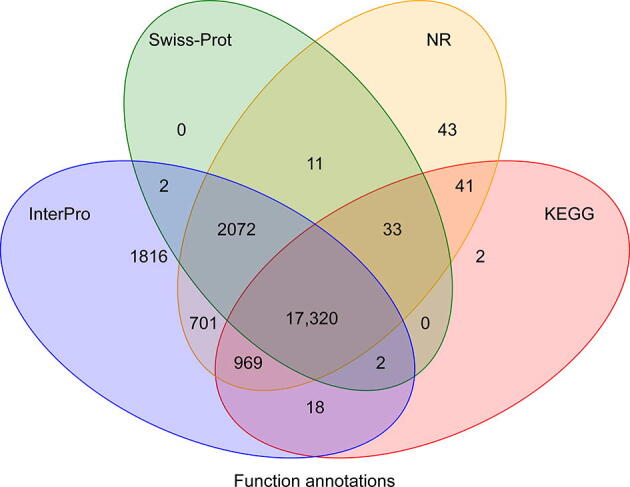
**Venn diagram of the function annotation for white-blotched river stingray’s protein-coding genes using four protein databases** NR, Non-Redundant Protein Sequence Database; KEGG, Kyoto Encyclopedia of Genes and Genomes.

The *P*. *leopoldi* genome assembly contains more than 71% repetitive content based on *de novo* and sequence homology analyses (Figure S1; Tables S6 and S7), which is much higher than that of white shark (58.5%) [12]. A total of 23,240 protein-coding genes were predicted with high-confidence by combining *de novo* and homologous gene prediction methods with the transcriptomic data and orthologs from other fish genomes (Table S8). This gene number seems higher than that of elephant shark but lower than that in white shark [1], [12]. We assigned preliminary functions to 23,030 (99.1%) protein-coding genes using BLASTp against protein databases including Swiss-Prot, Non-Redundant Protein Sequence Database (NR), Kyoto Encyclopedia of Genes and Genomes (KEGG), and InterPro (Figure S2; Table S9), and also assigned Gene Ontology (GO) terms to 21,040 (90.5%) protein-coding genes (Table S9). In addition, the orthologs of these protein-coding genes were also analyzed in other fish species (Table S10). Moreover, non-coding genes including microRNAs (miRNAs), transfer RNAs (tRNAs), ribosomal RNAs (rRNAs), and small nuclearRNAs (snRNAs) are also identified accordingly (Table S11).

### ***P***. *leopoldi* split from bony fish about 381 MYA and has the slowest evolutionary rate among fish species

**Figure 1:**
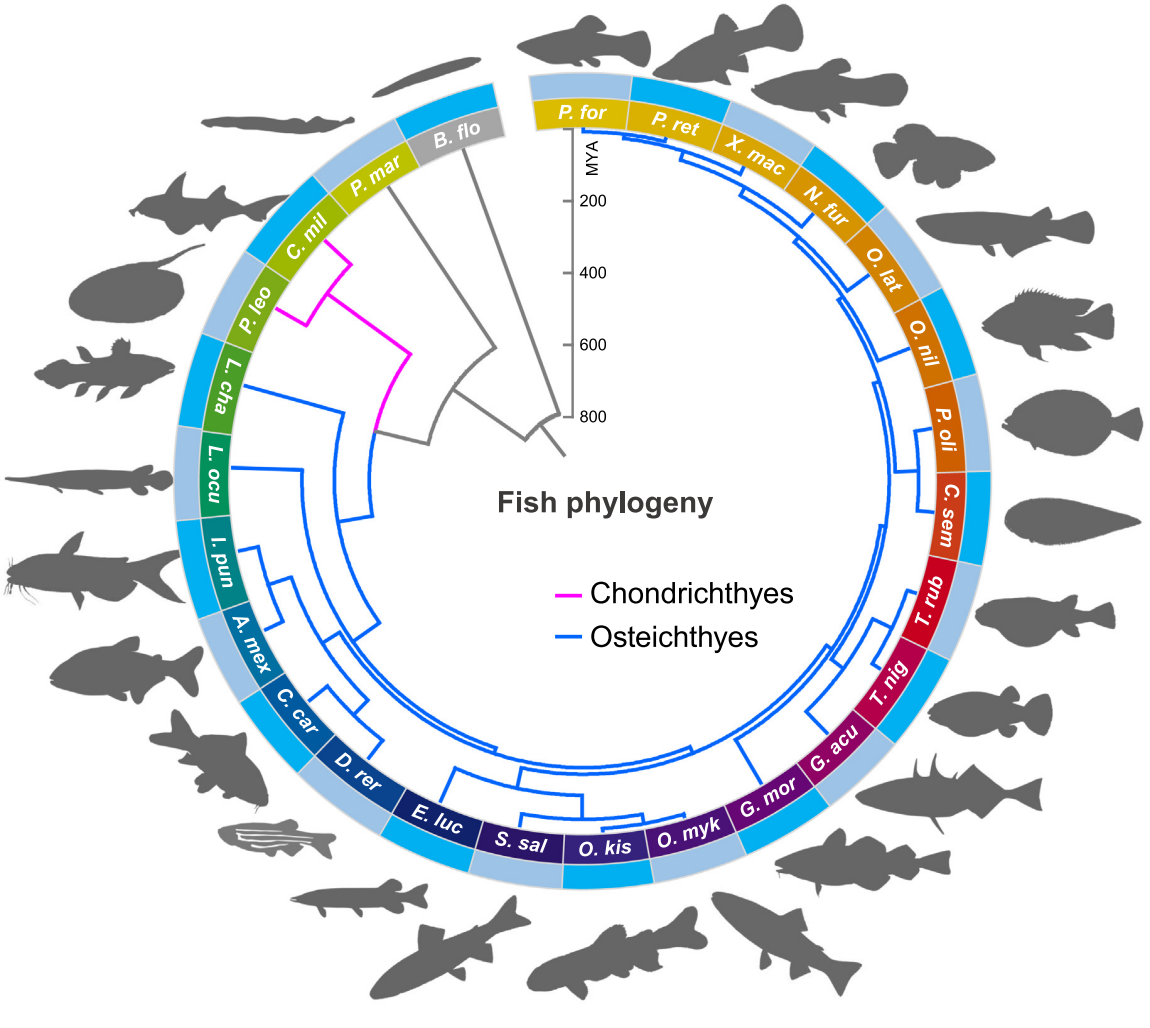
**Phylogeny of white-blotched river stingray and other 25 selected species**. The phylogenomic tree was constructed with FastTree and MRBAYES using 212 one-to-one orthologous genes. *B*. *flo* (amphioxus) was used to root the tree. The 25 fishes are *P*. *mar* (*Petromyzon* *marinus*; sea lamprey), *C*. *mil* (*Callorhinchus milii*; elephant shark), *P*. *leo* (*Potamotrygon leopoldi*; white-blotched river stingray), *L*. *cha* (*Latimeria chalumnae*; coelacanth), *L*. *ocu* (*Lepisosteus oculatu*; spotted gar), *I*. *pun* (*Ictalurus punctatus*; channel catfish), *A*. *mex* (*Astyanax mexicanus*; blind cave fish), *C*. *car* (*Cyprinus carpio*; common carp), *D*. *rer* (*Danio rerio*; zebrafish), *E*. *luc* (*Esox lucius*; northern pike), *S*. *sal* (*Salmo salar*; Atlantic salmon), *O*. *kis* (*Oncorhynchus kisutch*; coho salmon), *O*. *myk* (*Oncorhynchus mykiss*; rainbow trout), *G*. *mor* (*Gadus morhua*; Atlantic cod), *G*. *acu* (*Gasterosteus aculeatus*; three-spined stickleback), *T*. *nig* (*Tetraodon nigroviridis*; green spotted puffer), *T*. *rub* (*Takifugu rubripes*; Japanese puffer), *C*. *sem* (*Cynoglossus semilaevis*; tongue sole), *P*. *oli* (*Paralichthys olivaceus*; Japanese flounder), *O*. *nil* (*Oreochromis niloticus*; Nile tilapia), *O*. *lat* (*Oryzias latipes*; rice fish), *N*. *fur* (*Nothobranchius furzeri*; turquoise killifish), *X*. *mac* (*Xiphophorus maculatus*; platyfish), *P*. *ret* (*Poecilia reticulate*; guppy), and *P*. *for* (*Poecilia formosa*; Amazon molly). Magenta indicates the Chondrichthyes lineage and sky blue indicates the Osteichthyes lineage. Divergence time is shown in million years. MYA, million years ago.

**Supplementary Figure S3:**
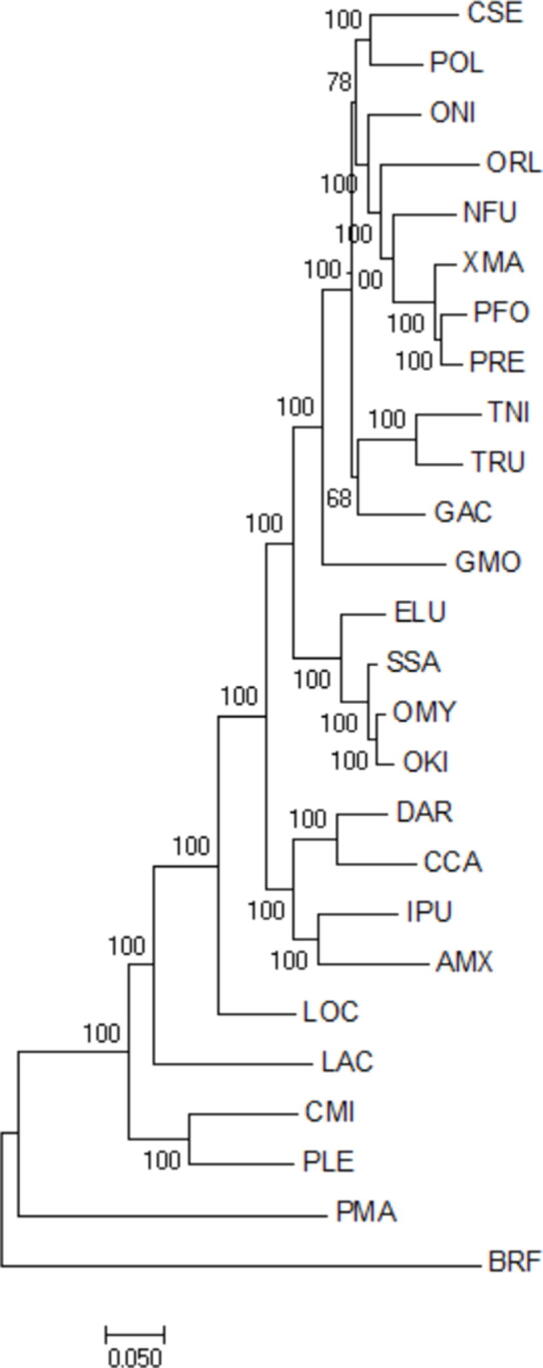
**The maximum likelihood tree of white-blotched river stingray and 25 selected species with statistical support values** AMX for blind cave fish. DAR for zebrafish. GMO for Atlantic cod. GAC for three-spined stickleback. LAC for coelacanth. LOC for spotted gar. ONI for Nile tilapia. ORL for rice fish. PMA for sea lamprey. PFO for Amazon molly. TRU for Japanese puffer. TNI for green spotted puffer. XMA for platyfish. CMI for elephant shark. CSE for tongue sole. CCA for common carp. ELU for northern pike. IPU for channel catfish. NFU for turquoise killifish. OKI for coho salmon. OMY for rainbow trout. POL for Japanese flounder. PRE for guppy. SSA for Atlantic salmon. BRF for amphioxus. PLE for white-blotched river stingray. Branch length is proportional to the substitution rate.

**Supplementary Figure S4:**
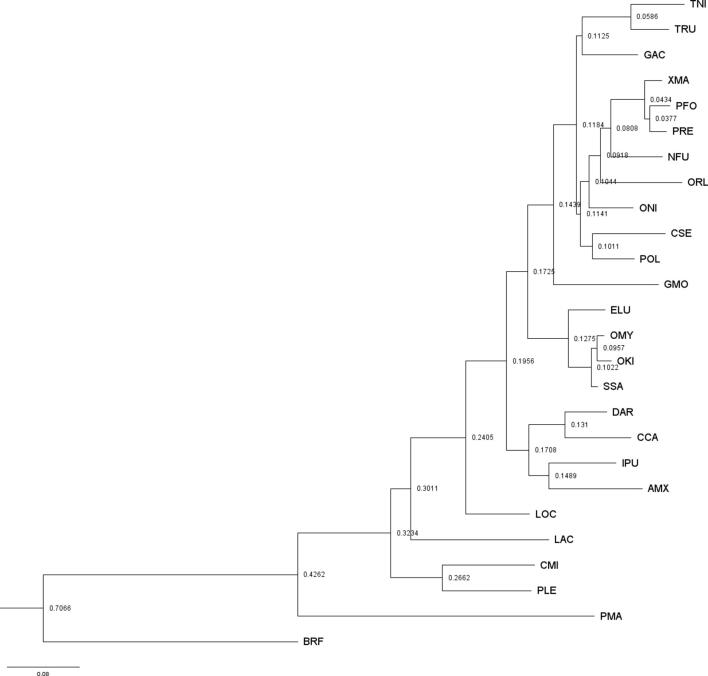
**The Bayesian tree of white-blotched river stingray and 25 selected species with posterior probability support** AMX for blind cave fish. DAR for zebrafish. GMO for Atlantic cod. GAC for three-spined stickleback. LAC for coelacanth. LOC for spotted gar. ONI for Nile tilapia. ORL for rice fish. PMA for sea lamprey. PFO for Amazon molly. TRU for Japanese puffer. TNI for green spotted puffer. XMA for platyfish. CMI for elephant shark. CSE for tongue sole. CCA for common carp. ELU for northern pike. IPU for channel catfish. NFU for turquoise killifish. OKI for coho salmon. OMY for rainbow trout. POL for Japanese flounder. PRE for guppy. SSA for Atlantic salmon. BRF for amphioxus. PLE for white-blotched river stingray. Branch length is proportional to substitution rate.

**Supplementary Figure S5:**
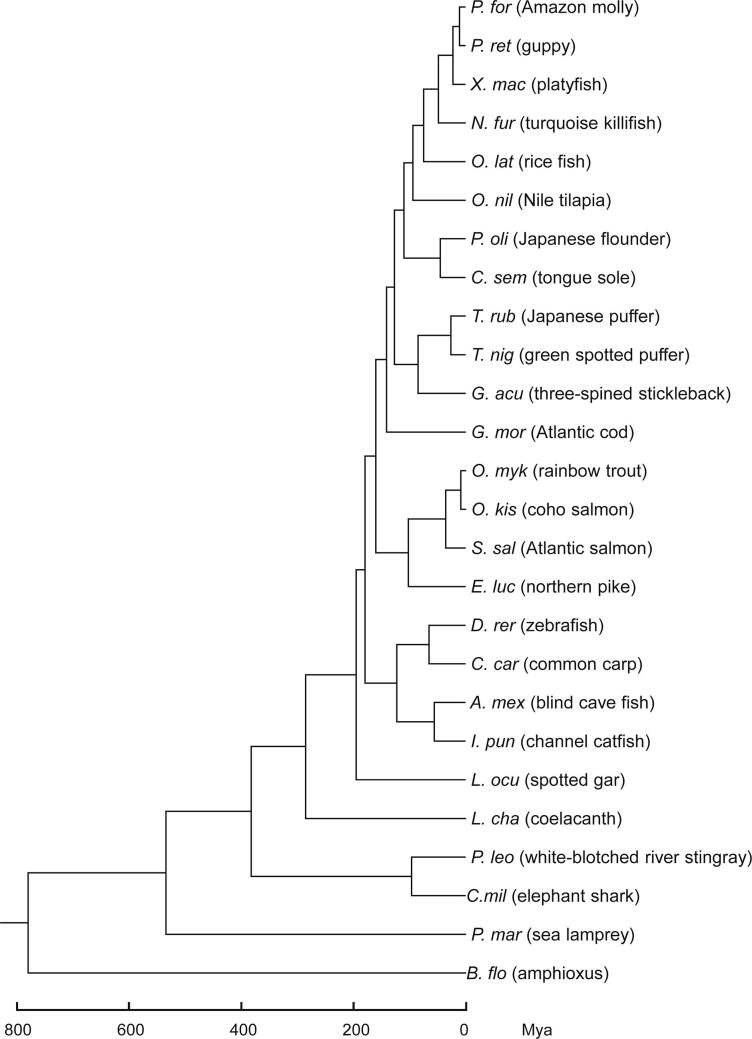
**The species tree of 25 selected fishes and one chordate**

**Supplementary Figure S6:**
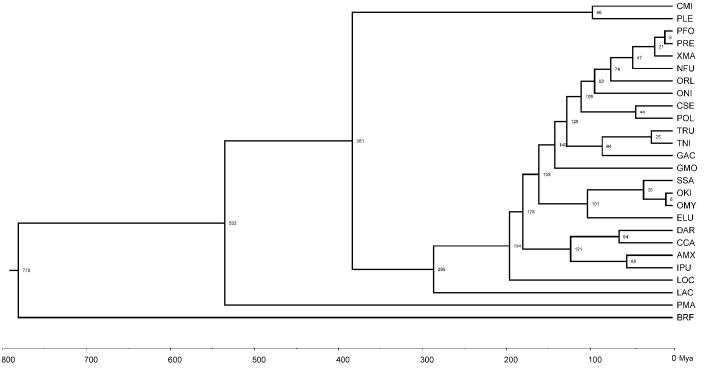
**The MCMCtree for estimating the divergence time of white-blotched river stingray and 25 selected species** AMX for blind cave fish. DAR for zebrafish. GMO for Atlantic cod. GAC for three-spined stickleback. LAC for coelacanth. LOC for spotted gar. ONI for Nile tilapia. ORL for rice fish. PMA for sea lamprey. PFO for Amazon molly. TRU for Japanese puffer. TNI for green spotted puffer. XMA for platyfish. CMI for elephant shark. CSE for tongue sole. CCA for common carp. ELU for northern pike. IPU for channel catfish. NFU for turquoise killifish. OKI for coho salmon. OMY for rainbow trout. POL for Japanese flounder. PRE for guppy. SSA for Atlantic salmon. BRF for amphioxus. PLE for white-blotched river stingray. The numbers adjacent to the nodes are divergence time which is shown in million years.

A phylogenomic tree of *P*. *leopoldi* and 24 other fish species was constructed using 212 one-to-one orthologous genes with 48,202 amino acid sites, with *B*. *floridae* (a chordate) as the outgroup. A jawless fish, sea lamprey (*Petromyzon marinus*), was basal to cartilaginous and bony fishes, and there was a conspicuous split between cartilaginous and bony fishes (Figure 1). Both maximum likelihood and Bayesian trees showed exactly the same topology (Figures S3 and S4). *P*. *leopoldi* was grouped with *C*. *milii*, forming the Chondrichthyes clade, whereas 22 other bony fishes were clustered as the Osteichthyes clade (Figure S5). This result is consistent with the traditional taxonomic classification of fishes. A MCMCtree-based divergence time estimation indicated that the split of the other fish species from the class Cyclostomata (*P*. *marinus*) occurred ∼ 533 MYA (Figure 1, Figure S6), and the splits between Chondrichthyes and Osteichthyes and between the superorder Batoidea (*P*. *leopoldi*) and Selachimorpha (*C*. *milii*) are ∼ 381 MYA and ∼ 96 MYA, respectively.

We also calculated the evolutionary rates as total substitution rate per site for each species, using the same set of orthologous genes as in our phylogenomic analysis (Table 2). *P*. *leopoldi* had the lowest amino acid substitution rate among 26 species. Tajima’s relative rate tests confirmed that its evolutionary rate was significantly slower than those of *B*. *floridae*, *P*. *marinus*, *L*. *chalumnae*, and *D*. *rerio*, but similar to the other cartilaginous fish, such as *C*. *milii* (Table S12). However, Tajima’s relative rate tests also showed that *L*. *oculatus* exhibited a slower evolutionary rate than *P*. *leopoldi* when using amphioxus or sea lamprey as the outgroup. Thus, the results above suggest that *P*. *leopoldi* is one of the slowest evolving fishes.

**Table 2:** **Amino acid substitution rate in 26 fish species**

**Species**	**Number of substitutions per site**
Amphioxus	0.408705
Sea lamprey	0.277635
Elephant shark	0.252635
White-blotched river stingray	0.250005
Coelacanth	0.265845
Spotted gar	0.250345
Channel catfish	0.338655
Blind cave fish	0.364285
Zebrafish	0.329255
Common carp	0.353285
Northern pike	0.329085
Atlantic salmon	0.321695
Rainbow trout	0.328565
Coho salmon	0.335825
Atlantic cod	0.381235
Three-spined stickleback	0.363065
Green spotted puffer	0.410095
Japanese puffer	0.394355
Nile tilapia	0.358895
Rice fish	0.407615
Turquoise killifish	0.388785
Platyfish	0.388865
Amazon molly	0.397335
Guppy	0.393995
Tongue sole	0.391035
Japanese flounder	0.359955

### Specific genes/expanded gene families found in ***P***. ***leopoldi***

**Figure 2:**
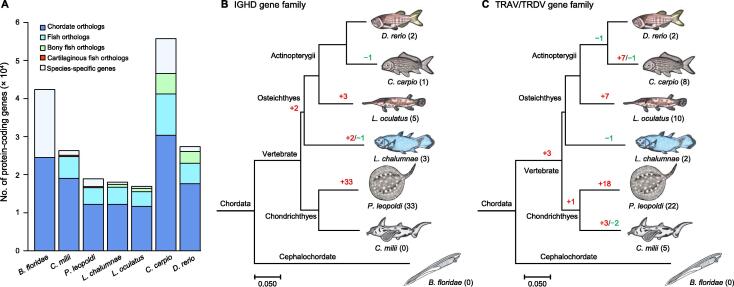
**Gene orthology and family analyses for seven species**. **A.** Gene orthology comparison among *B*. *floridae* (amphioxus), *C*. *milii* (elephant shark), *P*. *leopoldi* (white-blotched river stingray), *L*. *chalumnae* (coelacanth), *L*. *oculatus* (spotted gar), *C*. *carpio* (common carp), and *D*. *rerio* (zebrafish). **B.** Expansion and contraction of the IGHD gene family among seven species. **C.** Expansion and contraction of the TRAV/TRDV gene family among seven species. Number in red indicates the number of expanded genes; number in green indicates the number of contracted genes; number in the parenthesis indicates the total number of expanded and contracted genes. IGHD, immunoglobulin heavy constant delta; TRAV/TRDV, T-cell receptor alpha/delta variable.

To explore the evolutionary features of *P*. *leopoldi*, we performed an orthology analysis, by comparing its protein-coding genes with those of *B*. *floridae*, *C*. *milii*, *L*. *chalumnae*, *L*. *oculatus*, *C*. *carpio*, and *D*. *rerio* (Figure 2A). After filtering the genes encoding proteins shorter than 100 amino acids and having low sequence complexity from the predicted 23,240 protein-coding gene pool, we kept a total of 18,894 stingray protein-coding genes for orthology analysis. Among these kept *P*. *leopoldi* genes, 12,219, 4347, and 292 of them are chordate orthologs (shared with those of *B*. *floridae*), bony fish orthologs (shared with those of 5 other bony fishes), and cartilaginous fish orthologs (shared with those of *C*. *milii*), respectively. In addition, 2036 of them are *P*. *leopoldi*-specific genes (no homologous relationship with the other six species). Notably, *C*. *carpio* has four rounds of genome duplication and possesses a very large number of protein-coding genes (55,756) [13].

**Supplementary Figure S7:**
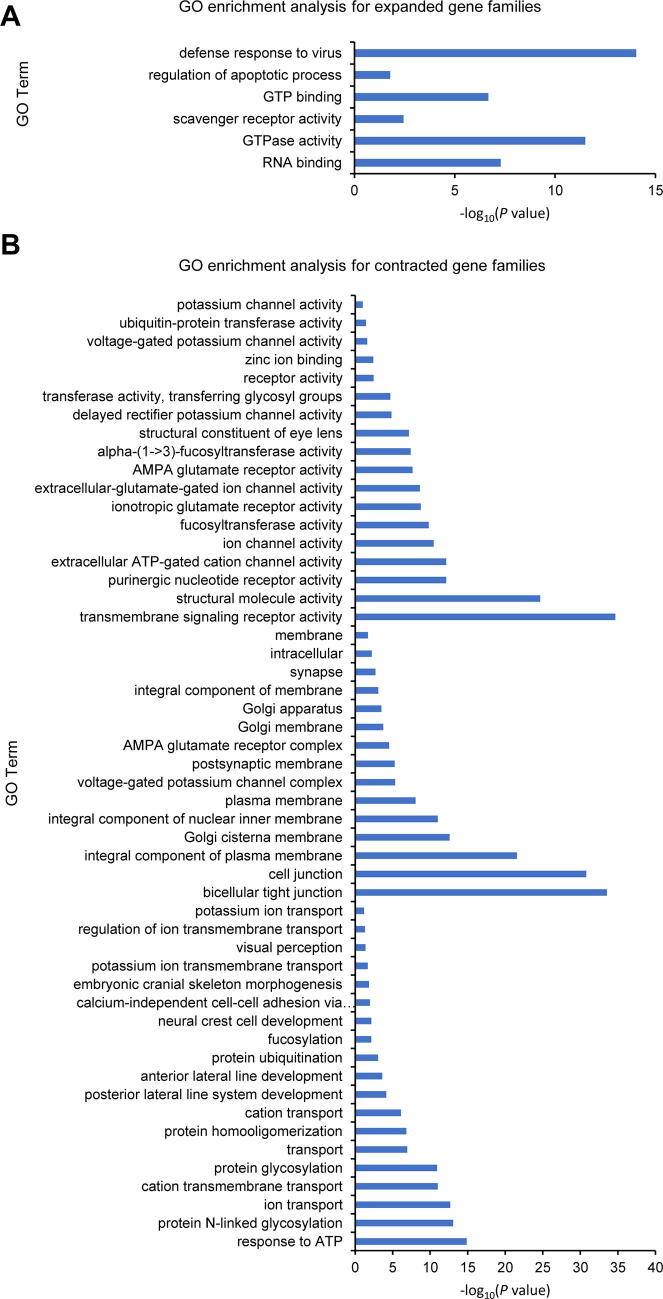
**GO enrichment analyses for expanded and contracted gene families identified in white-blotched river stingray** GO, Gene Ontology.

Next, we examined gene family expansions and contractions across seven selected species using a total of 205,728 protein-coding genes. According to their homologous relationships, these genes could be classified into 14,818 gene families, and 7830 out of these gene families experienced multiple expansion or contraction events in one or several selected species. A total of 56 gene families were significantly expanded and 14 families were significantly contracted in *P*. *leopoldi* only. GO analysis showed that the expanded and contracted gene families in  *P*. *leopoldi* had different biological emphases (Figure S7). The expanded gene families were primarily related to the immune system like defense response to virus (Bonferroni corrected *P* < 0.05), whereas the contracted gene families were more enriched with cellular component such as crystallin and lectin (Bonferroni corrected *P* < 0.05). Among the *P*. *leopoldi* gene families that experienced expansion, two of them, immunoglobulin heavy constant delta (IGHD) gene family and T-cell receptor alpha/delta variable (TRAV/TRDV) gene family, are important constituents of the vertebrate immune system (Figure 2B and C). Interestingly, their expansions were *P*. *leopoldi*-specific and not found in elephant shark, suggesting their possible roles in freshwater niche adaption. They were also under purifying selection tested with the branch model in PAML (data not shown).

### Eleven ***Hox*** genes are missing in ***P***. ***leopoldi*** Hox gene clusters

**Figure 3:**
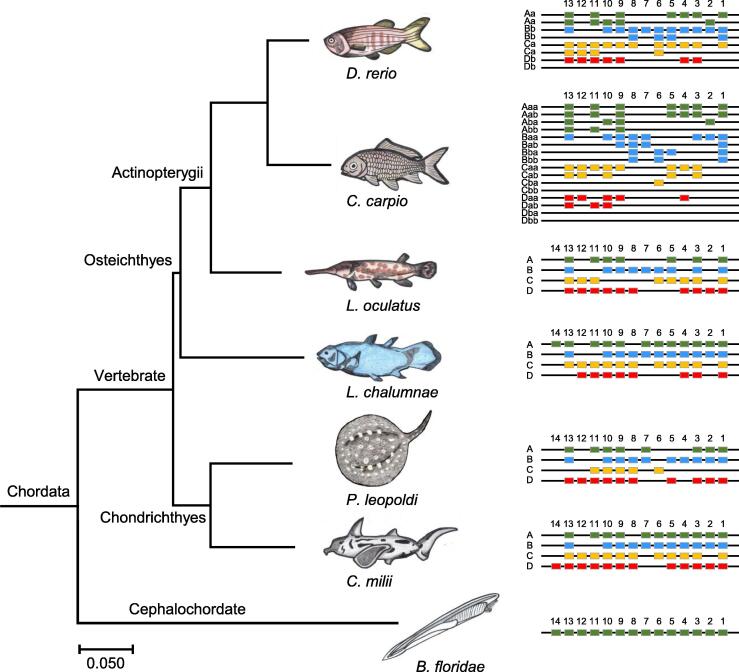
**Comparison of Hox gene clusters among seven fish species**. The *Hox* genes were identified from *B*. *floridae* (amphioxus), *C*. *milii* (elephant shark), *P*. *leopoldi* (white-blotched river stingray), *L*. *chalumnae* (coelacanth),  *L*. *oculatus* (spotted gar), *C*. *carpio* (common carp), and *D*. *rerio* (zebrafish).

The distinct body shape of *P*. *leopoldi* is assumed to have genetic basis, attributable to its Hox gene clusters that exhibit striking spatial collinearity and drive morphologic diversification of almost all metazoans. Due to the contribution of whole-genome duplication events among vertebrates (especially in teleost fishes) and lineage-specific secondary losses, the number of Hox gene clusters or genes varies greatly among vertebrates [14]. As shown in Figure 3, Hox gene clusters range from one in cephalochordate *B*. *floridae* to 13 in *C*. *carpio* in number [15], [16], and there are 33 *Hox* genes belonging to four putative Hox clusters (A, B, C, and D) in *P*. *leopoldi* within single scaffolds. Therefore, *P*. *leopoldi* retains the majority of 2R Hox cluster duplicates. Compared with *C*. *milii*, *P*. *leopoldi* possesses fewer *Hox* genes, especially in the HoxC cluster. Totally, *P. leopoldi* lacks *HoxA4*, *HoxA5*, and *HoxA6* in the HoxA cluster, *HoxB6* in the HoxB cluster, *HoxC1*, *HoxC3*, *HoxC4*, *HoxC5*, *HoxC12*, and *HoxC13* in the HoxC cluster, and *HoxD4* in the HoxD cluster. This difference in *Hox* gene composition may contribute greatly to stingray’s body shape differences compared with *C*. *milii*. Moreover, there are seven *Hox* genes lost in *P*. *leopoldi* but present in all other fishes, including *HoxA5*, *HoxB6*, *HoxC3*, *HoxC4*, *HoxC5*, *HoxC13*, and *HoxD4*. We thus assume that such *Hox* gene diversity between *P*. *leopoldi* and other fish species may genetically explain its specific body morphology.

### Diversified tissue expression profiles in ***P***. ***leopoldi***

**Figure 4:**
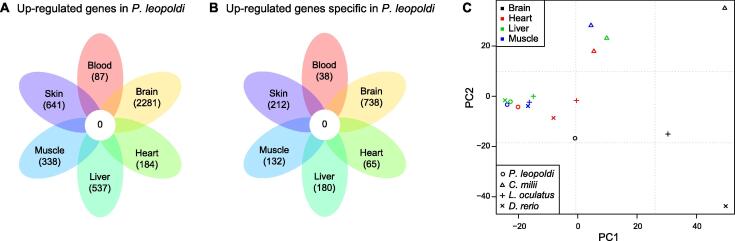
**Transcriptome analyses of*****P*****.*****leopoldi*****and other three fishes**. **A.** Venn diagram flower plot of up-regulated genes in six tissues of *P*. *leopoldi*. **B.** Venn diagram flower plot of up-regulated genes specific in *P*. *leopoldi* compared to *C. milii*, *L. oculatus*, and *D. rerio*. **C.** PCA of the expression levels of 3738 one-to-one orthologous genes in the brain, heart, liver, and muscle of *P.* *leopoldi*, *C*. *milii*, *L*. *oculatus*, and *D*. *rerio.* PC, principal component; PCA, principal component analysis.

**Supplementary Figure S8:**
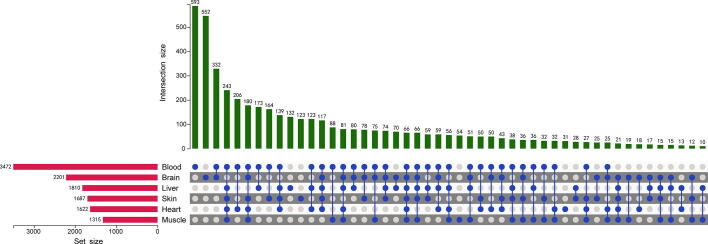
**Upset plot of down-regulated genes in six tissues of white-blotched river stingray**

To document its tissue-associated genes and evaluate their possible functions, we acquired RNA sequencing (RNA-seq) data from six *P*. *leopoldi* tissues. First, we identified each tissue’s differentially expressed genes (DEGs) by comparing its expression profile with the rest five ones. After normalization based on transcripts per kilobase million (TPM), 3559, 4482, 1806, 2347, 1703, and 2328 DEGs were identified in the blood, brain, heart, liver, muscle, and skin of *P*. *leopoldi*, respectively. Among these DEGs, 87, 2281, 184, 537, 388, and 641 genes were up-regulated, whereas 3472, 2201, 1622, 1810, 1315, and 1687 were down-regulated in the blood, brain, heart, liver, muscle, and skin, respectively. Up-regulated DEGs were all tissue-specific, and no shared up-regulated genes were found in the brain, heart, liver, muscle, and skin (Figure 4A). GO analysis showed that each tissue’s up-regulated DEGs were faithful to their tissue’s function (Table 3). In the six tissues of *P*. *leopoldi*, down-regulated DEGs shared many overlapping parts among six tissues, and exhibited a similar expression background of antibiotics biosynthesis, catalytic activity, and metabolic process (Figure S8). Compared to *C*. *milii*, *L*. *oculatus*, and *D*. *rerio*, about one third of the up-regulated DEGs in each tissue were *P*. *leopoldi*-specific (38/87 in blood, 738/2281 in brain, 65/184 in heart, 180/537 in liver, 132/388 in muscle, and 212/641 in skin), and thus their possible functions remain to be discovered in stingray (Figure 4B).

**Table 3:** **GO enrichment analysis of up-regulated tissue-specific genes in*P*. *leopoldi***

**Category**	**GO term**	***P* value**
**Blood**		
GOTERM_BP_DIRECT	Erythrocyte development	6.62 × 10^−6^
GOTERM_BP_DIRECT	Embryonic hemopoiesis	3.41 × 10^−5^
GOTERM_BP_DIRECT	Erythrocyte differentiation	6.48 × 10^−5^
GOTERM_BP_DIRECT	Hemopoiesis	7.09 × 10^−4^
**Brain**		
GOTERM_BP_DIRECT	Central nervous system development	8.02 × 10^−4^
INTERPRO	Neurotransmitter-gated ion-channel	0.001797
GOTERM_BP_DIRECT	Ganglioside biosynthetic process	0.002529
GOTERM_BP_DIRECT	Neuropeptide signaling pathway	0.012035
GOTERM_BP_DIRECT	Neural crest cell migration	0.016101
GOTERM_BP_DIRECT	Hindbrain development	0.027042
**Heart**		
GOTERM_BP_DIRECT	Cardiac muscle cell differentiation	2.46 × 10^−5^
GOTERM_BP_DIRECT	Heart morphogenesis	6.17 × 10^−4^
GOTERM_BP_DIRECT	Heart looping	9.05 × 10^−4^
GOTERM_BP_DIRECT	Heart development	0.005143
**Liver**		
GOTERM_BP_DIRECT	Liver development	7.39 × 10^−4^
GOTERM_MF_DIRECT	Oxidoreductase activity	1.54 × 10^−11^
KEGG_PATHWAY	Glycine, serine and threonine metabolism	0.007413
KEGG_PATHWAY	Fatty acid degradation	8.55 × 10^−5^
**Muscle**		
GOTERM_BP_DIRECT	Muscle organ development	1.76 × 10^−5^
GOTERM_BP_DIRECT	Skeletal muscle tissue development	7.32 × 10^−5^
GOTERM_BP_DIRECT	Myofibril assembly	2.78 × 10^−4^
INTERPRO	Myogenic basic muscle-specific protein	4.65 × 10^−4^
GOTERM_BP_DIRECT	Skeletal muscle cell differentiation	0.00223
UP_KEYWORDS	Myogenesis	0.003216
**Skin**		
GOTERM_BP_DIRECT	Ectodermal placode formation	0.068466
GOTERM_CC_DIRECT	Melanosome	0.015904

Next, we compared the expression profiles of 3738 one-to-one orthologs in four tissues (brain, heart, liver, and muscle) between *P*. *leopoldi*, *C*. *milii*, *L*. *oculatus*, and *D*. *rerio*, and performed the principal component analysis (PCA) to investigate the expression patterns for the four tissues across the four fishes. The PCA result showed a scattered expression pattern for each of the four tissues across four fishes (Figure 4C). Our data were separated neither by tissue nor by species. In mammals, expression analyses have shown that the same tissues from different species tend to cluster together [17], proposing that the same tissues in different mammals usually perform similar physiological functions. In fishes, the diverged expression pattern of the same tissues suggests a probably much more diversified physiology or a much longer evolutionary history, both of which are not mutually exclusive.

### ***P***. ***leopoldi*** lacks the ***gc*** gene for hard skeleton formation

**Figure 5:**
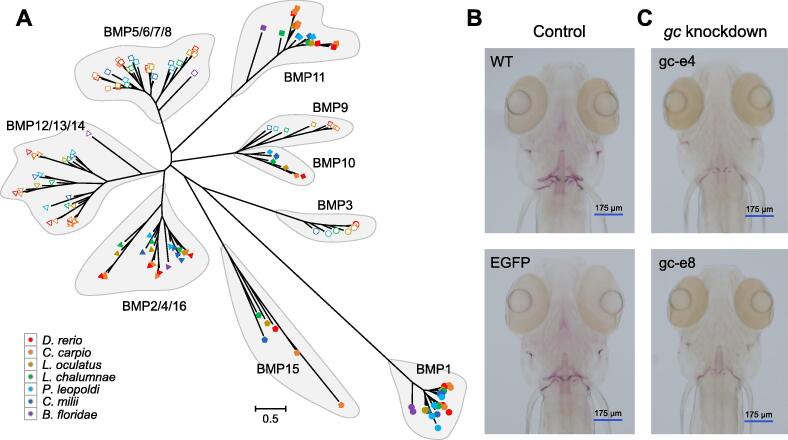
**Analyses of bone formation-related genes among seven selected species**. **A.** Phylogenetic analysis of BMP gene family among seven selected species. **B.** Alizarin Red S staining of 6-dpf control zebrafish. WT represents embryos without injection; EGFP represents embryos injected with RNPs against *EGFP*. **C.** Alizarin Red S staining of 6-dpf *gc*-knockdown zebrafish. gc-e4 represents embryos injected with RNPs against exon 4 of the *gc* gene; gc-e8 represents embryos injected with RNPs against exon 8 of the *gc* gene. WT, wild-type; dpf, days post-fertilization; BMP, bone morphogenetic protein; RNP, ribonucleoprotein; EGFP, enhanced green fluorescent protein.

As a cartilaginous fish, *P*. *leopoldi* already has a complex skeleton structure providing support for its body and internal organs. How hard skeleton emerged in vertebrates and what is the genetic basis for bone formation remain to be investigated. We systematically compared the bone formation-related gene families between cartilaginous and bony fish genomes. First, we examined the bone morphogenetic protein (BMP) gene family and BMP receptor (*BMPR*) genes among seven selected species. It is known that both BMPs and their receptors play an essential role in skeleton formation. Generally, *BMP* genes are classified into eight different clusters according to their phylogenetic relationships (Figure 5A). Cartilaginous and bony fishes have the representative genes in all eight clusters. *P*. *leopoldi* does not have the *bmp15* gene, whereas *C*. *milii* has. Compared with fish species, *B*. *floridae* does not have the *bmp3*, *bmp9*, *bmp10*, *bmp15*, and *bmp16* genes. As to *BMPR* genes [18], both types I and II are present in both cartilaginous and bony fishes, but *B*. *floridae* misses *BMPR* type II (Table S13). Our analyses suggest that BMPs and their receptors are less likely to answer the question whether the skeleton is made of cartilage or hard bone, because both cartilaginous and bony fishes have all representative *BMP* and *BMPR* genes. Second, we examined the presence or absence of other bone formation-related genes between cartilaginous and bony fishes. After scrutinizing the candidate gene list involved in skeleton formation from six fish species, we found that the *gc* gene, encoding vitamin D-binding protein (VDBP), is present in bony fishes but absent in cartilaginous fishes. *L*. *chalumnae*, *L*. *oculatus*, *C*. *carpio*, and *D*. *rerio* have 2, 1, 3, and 1 copies of *gc*, respectively. We hypothesize that VDBP may be involved in the bone formation process for bony fishes. *D*. *rerio* is a widely used model organism for studies of bone development and formation [19]. To verify the possible function of the *gc* gene in bone formation, we designed two small guide RNAs (sgRNAs), gc-e4 and gc-e8, against the exon 4 and exon 8 of the *gc* gene, respectively [20], using  a sgRNA against the enhanced green fluorescent protein (*EGFP*) gene as a scrambled control. As shown in Figure 5B and C, embryos injected with control ribonucleoproteins (RNPs) displayed normal bone formation, whereas embryos injected with RNPs against the *gc* gene displayed incomplete craniofacial skeleton mineralization. The results support our hypothesis that the *gc* gene is involved in hard skeleton formation.

## Discussion

### The evolutionary features of the genome and Hox gene clusters of *P*. *leopoldi*

The genomic data of *P*. *leopoldi* provide us an information bonanza for understanding fish evolution, especially the split between cartilaginous and bony fishes. Our phylogenomic analysis showed that Chondrichthyes and Osteichthyes were two parallel monophyletic groups and the split between them can be dated back to 381 MYA, which is less than the estimation of 450 MYA based on mitochondrial genome [3]. The discrepancy between our result and the mitogenomic estimation could be caused by the different evolutionary rates of nuclear genome and mitochondrial genome. Mitochondrion has a particularly high mutation rate and a much diversified evolutionary spectrum across species [21]. The non-unified estimations of split time can be explained by different data sources. Generally, the evolutionary rate of elasmobranchs is much lower than that in mammals [22], [23], and the Chondrichthyes also shows a slower evolutionary rate than the Osteichthyes. In this study, *P*. *leopoldi*, *C*. *milii*, and two basal bony fish species (*L*. *chalumnae* and *L*. *oculatus*) evolved much slower than the other fish species (Figure 1). These four fish species also did not experience the third round of genome duplication (3R) which happened in the ray-finned fish lineage [24], [25]. This result supports the hypothesis that the 3R might promote the teleost in a higher rate of sequence evolution [1], [26], [27], and suggests that cartilaginous and ancient bony fishes might have been well adapted to their niches. In *P*. *leopoldi*, *C*. *milii*, *L*. *chalumnae*, and *L*. *oculatu*, the substratum (the number of genes) for evolution to work on was limited and therefore slow evolution is expected. Although *P*. *leopoldi* genome was not assembled at the chromosomal level, the completeness of the genome has achieved 91.8% coverage estimated by the BUSCO methoed. *P*. *leopoldi* has four Hox gene clusters containing 33 genes, the smallest number of *Hox* genes among six fish species analyzed in our study (Figure 3). *C*. *milii*, *L*. *chalumnae*, *L*. *oculatus*, *C*. *carpio*, and *D*. *rerio* have 45, 42, 34, 62, and 49 *Hox* genes, respectively. Moreover, *P*. *leopoldi* lacks *HoxA5*, *HoxB6*, *HoxC3*, *HoxC4*, *HoxC5*, *HoxC13*, and *HoxD4* presented in the other five fish species. *P*. *leopoldi* has a disc-like body, whereas most fish species usually have a streamline body. The *Hox* gene difference between *P*. *leopoldi* and the other fish species may help to explain why *P*. *leopoldi* has a simple body design with large pectoral fins and a whip-like tail. However, our study does not provide the answer for which *Hox* gene contributes to what morphological alteration in stingray’s body plan.

### The expansion of two immune-related gene families in *P*. *leopoldi*

Our gene family analysis showed that 56 gene families were significantly expanded in *P*. *leopoldi*. Many of them encode protiens involved in immune and stress response, such as heat shock proteins (HSPs), cytochrome P450 (CYP450), and inhibitors of apoptosis (IAPs). Two expanded gene families, IGHD and TRAV/TRDV, were found to be directly related to the immune system (Figure 2). Immunoglobulins are membrane-bound or secreted glycoproteins produced by B lymphocytes. Immunoglobulin D (IgD) is made up of two heavy chains of the delta class, and two light chains. IgD is present in species ranging from fish to mammal, suggests that IgD has important immunological functions. IgD has been reported to be able to bind to basophils and mast cells and induce antimicrobial factors, which contributes to immune surveillance and inflammation under pathological conditions [28]. T-cell receptor alpha (TCRα) recognizes peptides that are bound to major histocompatibility complex (MHC) molecules, and TCR delta (TCRδ) recognizes antigens directly, both of which are considered as a bridge between the innate and adaptive immune system. TCRα and TCRδ cDNA sequences have been identified in the nurse shark [29], as well as in other vertebrates. The existence of TCRs in sharks suggests that the adaptive immune system evolved in cartilaginous fish has the same fundamental major components as those existing in vertebrates such as humans [30]. Our studies of TCR expansion in *P. leopoldi* help elucidate that the *V* genes of TCRs have evolved for 500 million years, and indicate their role in the diversification of the pre-immune repertoire. The natural habitat of *P*. *leopoldi* is Xingu River basin, and the phylogenetic relationship of stingrays shows that the family Potamotrygonidae evolved from the sea-dwelling ancestors [31]. The fact that stingrays can transit from marine environment to pathogen rich freshwater suggests that stingrays should have a complex immune system. The expansion of immune system-related genes (Figure 2) suggests that *P*. *leopoldi* could require specific immune responses for pathogens and antigens in new niches, although there has been no evidence that these two gene families are directly involved in freshwater habitat adaptation. Usually, genes related to immune response are evolutionarily active to coup with the constant-evolving pathogens [32]. When a gene family expanded through duplication event, some duplicated genes would be free from their function constraint. The old genes performed the normal function, and the new one would go through the neo-functionalization or sub-functionalization process [33]. Thus, their selection pressure tends to be positive, as well. However, our selective pressure analysis showed that these two gene families were under negative selection, a sign of function constraint. It indicates that their functions were rapidly fixed after the gene family expansion. One possible explanation for this phenomenon is that *P*. *leopoldi* fast adapted to its new niche in Xingu River and its immune system had to rapidly deal with new enemy after its ancestor migrated from the Atlantic Ocean to the Amazon River system. Furthermore, the fact that *P*. *leopoldi* no longer migrated to the other niches renders the functions of these expanded genes important for its survival in order to deal with fixed microbial enemy. Thus, these two expanded gene families were negatively selected. Surely, the argument mentioned above is our hypothesis and needs to be verified in the other fish species whose ancestor migrated to river from ocean.

### The gene expression silhouette of ***P***. ***leopoldi*** tissues

Expression profile comparison among six *P*. *leopoldi* tissues demonstrates that its brain has a much wider expression spectrum than the other tissues (Figure 4), and the result is consistent with that of mammalian organs. In mammals, the central nervous system has more specifically expressed genes than the heart and liver [34]. Compared with mammals, there are more species-specific genes expressed in *P*. *leopoldi* in each of the analyzed tissues [17]. The brain-specific modules are enriched with genes involved in typical processes as for central nervous system development (Benjamini–Hochberg corrected *P* < 0.05), and thus define common neural tissue functions. Furthermore, the same tissues from different fish species exhibited a much more diversified expression pattern than that observed in mammals. The earliest mammal appeared around 225 MYA, and our analysis showed that fishes emerged at least 533 MYA [35]. As fishes have a much longer evolutionary history than mammals, a greater divergence for fishes at both expression and sequence levels is expected. We are only able to identify 3738 one-to-one orthologous genes among white-blotched river stingray, elephant shark, coelacanth, and zebrafish, which is much less than 5636 amniotic one-to-one orthologous genes identified among nine endothermic species in a previous study [17]. Therefore, the long fish evolutionary history may explain the non-unified expression profiles of their tissues at least in part. Our analysis of tissue transcriptomes from all representative fish lineages refines previous hypotheses and provides a new viewpoint for the evolution of chordate tissue functions.

### Absence of VDBP in *P*. *leopoldi*

Skeleton is an essential part of vertebrate body, which can be made of hard bone or cartilage only. For bone, two cell types, osteoblast and osteocyte, contribute to the formation and mineralization, but cartilage has only one cell type called chondrocyte [18], [19]. Therefore, the emergence of hard skeleton in vertebrates must have engaged complex cellular processes and multiple genetic inventions. We searched the *P*. *leopoldi* gene inventory for the ones known to be involved in bone formation in osteichthyans. All gene families involved in skeleton formation seem to be present, except the *gc* gene which encodes VDBP. Our analyses of *BMP* and *BMPR* genes show that both cartilaginous and bony fishes have all representative members of these two gene families (Figure 5A; Table S13). The slight difference in *BMP* and *BMPR* genes between the two fish lineages is insufficient to explain the major departures in their skeletons. One major difference between bones and cartilages is whether calcium phosphate is present in their extracellular matrix or not. A previous study has reported that secreted calcium-binding phosphoproteins (SCPP) are involved in the bone formation in *D*. *rerio* [1]. The analysis of *C*. *milii* genome has reported the lack of genes encoding SCPP in cartilaginous fishes, which explains the absence of bone in their endoskeleton [1]. Our study further demonstrates that VDBP may also take part in the bone formation process (Figure 5B and C). Vitamin D promotes the absorption of calcium through the intestines [36]. Together, our result and that of a previous study both suggest that genes responsible for calcium metabolism are essential for the hard skeleton formation in bony fishes. Because cartilaginous and bony fishes evolved in parallel, several crucial genes alone may sufficiently exclude the hard skeleton from cartilaginous fishes, albeit under ongoing studies.

## Conclusion

In this study, we report the assembly and analysis of a draft genome of *P*. *leopoldi*, a cartilaginous freshwater fish. Because cartilaginous fishes constitute a critical outgroup for understanding the evolution and diversity of bony vertebrates, the whole-genome analysis shows that the *P*. *leopoldi* genome is evolving significantly slower than other vertebrates. The transcriptomic data shed lights into highly diversified gene expression profiles among fish lineages, as opposed to the coordinated gene expression among mammalian tissues. The expansion of immune-related gene families IGHD and TRAV/TRDV suggests that the diversification of the pre-immune repertoire in cartilaginous fishes could play a role in the evolution of an adaptive immune system. Our study further demonstrates that VDBP may partly explain the absence of hard bone in their endoskeleton. Together, our results starting from the *P*. *leopoldi* genome to the experimental model provide novel clues for niche adaptation and skeleton formation in the evolutionary history of fishes.

## Materials and methods

### *P*. *leopoldi* sample

A mature male *P*. *leopoldi* individual was acquired from an aquarium in China in January, 2018. It was the descendant of captive *P*. *leopoldi* breeding population. The fish was killed in a humane way, and the experimental procedure was performed in accordance with the guidelines of the Animal Care Committee at the Institute of Neuroscience, Shanghai Institutes for Biological Sciences, Chinese Academy of Sciences. Its skin, heart, blood, muscle, liver, and brain were used for DNA and RNA preparation and sequencing library construction.

### Genome sequencing and assembly

The genomic DNA of *P*. *leopoldi* was sequenced by WGS strategy. Based on the genome features, three different lengths (230 bp, 350 bp, and 450 bp) of DNA inserts were produced. The Illumina HiSeq2000 platform (San Diageo, CA) was used to sequence these reads by the paired-end sequencing method with the read length of 150 bp in order to capture the whole-genome data. A total of 17 DNA libraries were constructed, and the total amount of sequencing data was 882.2 Gb with a coverage of 200.95×. The PacBio SMRT platform yielding an average read length of 20 kb (Menlo Park, CA) was also used to generate 270.51-Gb data, equivalent to a genome coverage of 61.61×. Additionally, a 10X Genomics library was constructed, coupled with the Illumina sequencing platform in a read length of 150 bp, yielding 475.93-Gb data, equivalent to a genome coverage of 108.41×. PacBio long reads were utilized to perform *de novo* assembly. Around 31 million subreads were used for the assembly with FALCON (v0.3.0) to generate contigs [37]. Primary contigs were polished using Quiver5. The scaffolds were built based on 10X Genomics data. Sequence data were generated using the 10X Genomics GemCode platform (Pleasanton, CA), and the error-corrected contigs were used as input for scaffolding to obtain the primary assembly. After scaffolding, shotgun sequences were used to close gaps between contigs. Paired-end clean reads from the Illumina platform were aligned to the assembly with BWA [38]. Contigs or scaffolds shorter than 10 kb were excluded from the analysis to avoid spurious misassembly. Gaps in contigs and scaffolds were closed with subreads. To survey the characteristics of the genome, a total of ∼ 140-Gb next-generation sequencing data equivalent to a genome coverage of 33× were generated. Adaptor sequences, polymerase chain reaction (PCR) duplicates, and low-quality sequences were removed from the raw data to generate high-quality sequences. *K*-mer statistics of the high-quality sequences were calculated by Jellyfish (v2.2.7) with the parameters of “-G 2 -m 17” [39]. GenomeScope 2.0 (https://github.com/tbenavi1/genomescope2.0) [40] was used to estimate the size, heterozygosity rate, and repeat content of the *P*. *leopoldi* genome. Finally, the completeness of the assembly was assessed through BUSCO analysis (v5.2.1; https://busco.ezlab.org/) and CEG analysis (https://korflab.ucdavis.edu/Datasets/cegma/).

### Genome annotation

Repeat elements were annotated with both homology annotation and *de novo* prediction. RepeatMasker and RepeatProteinMask (https://www.repeatmasker.org/RepeatProteinMask.html) were used to search the assembled genome against Repbase for known repeat elements [41], [42]. LTR-FINDER and RepeatModeler (https://www.repeatmasker.org/RepeatModeler.html) were used to *de novo* develop repeat element library [43]. After the library was established, RepeatMasker was further used to detect species-specific repeat elements. Tandem repeats were also searched with Tandem Repeats Finder in the assembled *P*. *leopoldi* genome [44]. Overlapping transposable elements belonging to the same type of repeats were integrated together.

Protein-coding genes were predicted through combination of *de novo* annotation, homology annotation, and transcriptome-based annotation. AUGUSTUS, GlimmerHMM, SNAP (https://github.com/KorfLab/SNAP), Geneid, and GENSCAN (https://hollywood.mit.edu/GENSCAN.html) software packages were used to *de novo* predict protein-coding genes in the *P. leopoldi* genome [45], [46], [47]. For homology annotation, the protein sequences from Japanese puffer (*Takifugu rubripes*), rice fish (*Oryzias latipes*), Nile tilapia (*Oreochromis niloticus*), Atlantic cod (*Gadus morhua*), elephant shark (*C*. *milii*), green spotted puffer (*Tetraodon nigroviridis*), zebrafish (*D*. *rerio*), amphioxus (*B*. *floridae*), three-spined stickleback (*Gasterosteus aculeatus*), and coelacanth (*L*. *chalumnae*) were used to search the homologous genes in *P*. *leopoldi* genome with BLAST and GeneWise [48], [49]. For transcriptome-based annotation, the RNA-seq reads from *P*. *leopoldi* skin, heart, blood, muscle, liver, and brain were mapped and assembled with PASA and Cufflinks [50], [51]. EVidenceModeler (EVM) was employed to integrate the gene sets from three annotation methods into a complete and non-redundant gene set [52]. Finally, PASA was used to correct the EVM annotation result with untranslated region (UTR) and alternative splicing information.

Function annotation was performed through comparing the annotated protein-coding genes with the known protein banks. The final gene set was blasted against four common protein banks, Swiss-Prot (https://www.uniprot.org/), NR (https://www.ncbi.nlm.nih.gov/protein), KEGG (https://www.genome.jp/kegg/), and InterPro (https://www.ebi.ac.uk/interpro/). InterProScan was used to integrate the functional results from four protein banks [53].

The tRNA genes were identified by tRNAscan-SE software with eukaryote parameters [54]. The rRNA fragments were predicted by aligning to whale shark and *C*. *milii* template rRNA sequences using BLASTN at E-value of 1 × 10^–10^ [55]. The miRNA and snRNA genes were predicted using Infernal software by searching against the Rfam database (release 9.1) [56].

### Orthology analysis

We first compiled the complete proteomes of 25 fish and one chordate genomes. The proteome data of 13 selected organisms, including blind cave fish (*Astyanax mexicanus*), zebrafish (*D*. *rerio*), Atlantic cod (*G*. *morhua*), three-spined stickleback (*G*. *aculeatus*), coelacanth (*L*. *chalumnae*), spotted gar (*L*. *oculatus*), Nile tilapia (*O*. *niloticus*), rice fish (*O*. *latipes*), sea lamprey (*P*. *marinus*), Amazon molly (*Poecilia formosa*), Japanese puffer (*T*. *rubripes*), green spotted puffer (*T*. *nigroviridis*), and platyfish (*Xiphophorus maculatus*), were obtained from the Ensembl database (release 83, https://www.ensembl.org/). The proteome data of other 11 selected organisms, including elephant shark (*C*. *milii*), tongue sole (*Cynoglossus semilaevis*), common carp (*C*. *carpio*), northern pike (*Esox lucius*), channel catfish (*Ictalurus punctatus*), turquoise killifish (*Nothobranchius furzeri*), coho salmon (*Oncorhynchus kisutch*), rainbow trout (*Oncorhynchus mykiss*), Japanese flounder (*Paralichthys olivaceus*), guppy (*Poecilia reticulate*), and Atlantic salmon (*Salmo salar*), were downloaded from the National Center for Biotechnology Information (NCBI) database (https://www.ncbi.nlm.nih.gov/). The proteome of *B*. *floridae* was download from JGI Genome Portal (https://genome.jgi.doe.gov/portal/). Proteins shorter than 100 amino acids were discarded and, for alternatively spliced genes, only the longest splice variant of each gene was retained.

Orthologous protein groups were determined by OrthoFinder (v2.3.3) with blast search and default parameters [57]. This procedure led to 2792 orthologous groups with at least one representative protein from aforementioned 25 species plus *P*. *leopoldi*. To maximize orthology, these orthologous groups were filtered with an in-house Perl script to provide a subset group that contained strict one-to-one orthologous proteins from each species. We eventually retained 212 one-to-one orthologous groups for phylogenomic analysis.

### Phylogenomic analysis and estimation of divergence time

Protein sequences in each of the 212 one-to-one orthologous groups were aligned using MUSCLE and ClustalW, and the resulting alignments were combined by M-Coffee to produce the multiple sequence alignments (MSA) [58], [59], [60]. We used an in-house Perl script to remove gaps, and the final MSA contained 48,202 amino acid sites. FastTree 2.1 was used to construct the maximum likelihood tree for the final MSA with Jones–Taylor–Thornton (JTT) and category mixture model (CAT) models [61]. MRBAYES 3.2.6 was used to construct the Bayesian tree for the final MSA with JTT and invgamma models [62]. We ran the Markov chain Monte Carlo (MCMC) algorithm for 500,000 generations with 4 chains. Bayesian trees were sampled every 100 generations, and the first 25% of trees were excluded from the analysis as burn-in. The Bayesian tree was summarized after the average standard deviation of split frequencies below 0.01. RAxML was used to constructed a maximum likelihood tree using the JTT model with gamma distribution [63]. Then, MCMCtree was used to predict the divergence time of 26 species [64]. The intervals of the divergence time between different species were obtained from the TimeTree database [65].

### Identification of Hox gene clusters

Forty-nine unique *Hox* genes obtained from a previous study were used as queries to conduct BLAST search (threshold of E-value 1 × 10^−20^) against seven species, including *P*. *leopoldi*, *C*. *carpio*, *L*. *oculatus*, *L*. *chalumnae*, *D*. *rerio*, *C*. *milii*, and *B*. *floridae*, respectively [16], [48]. The Hidden Markov mode (HMM) profile for the homeodomain was used to identify the potential homeodomain containing genes from these genomes with HMMER 3.2.1 (https://hmmer.janelia.org/) [66], as well. All of the obtained genes were further validated using SMART database to determine whether the protein sequences contain homeodomains [67].

### Gene family expansion and contraction estimation and selective pressure analysis

Gene family expansion and contraction were analyzed by CAFE (v3.1) using the same seven species in the identification of Hox gene clusters [68]. After filtering the genes encoding proteins shorter than 100 amino acids and with low sequence complexity, we collected a total of 205,728 genes from seven selected species. OrthoFinder (v2.3.3) was used to classify these genes into orthologous groups based on their sequence similarity. Each orthologous group is actually a gene family. We calculated the probability of each orthologous group by 10,000 Monte Carlo random samplings and estimated the lambda value based on the maximum likelihood model, which represents the rate of expansion and contraction of each gene family. A branch with *P* < 0.05 was considered to have gene amplification and contraction over evolutionary time scales.

PAML was used to estimate the selective pressure of selected genes. Both branch and branch-site mode were applied to detected the selective pressure in *P*. *leopoldi* [64]. PAL2NAL was utilized for alignment nucleotide sequences based on protein alignment [69]. The likelihood ratio tests (LRTs) of M1a *vs.* M2a were employed to examine the selective pressure of each site among selected genes.

### Transcriptome analysis

Total RNA from six *P*. *leopoldi* tissues (skin, heart, blood, muscle, liver, and brain) was prepared using the Qiagen RNeasy Kit (Catalog No. 75142, Qiagen, Düsseldorf, Germany) according to the manufacturer’s instructions, and RNA-seq libraries were constructed according to a standard protocol for the Illumina novaseq6000 sequencing platform (San Diageo, CA) with 100-bp paired-end reads. The reads were aligned onto the assembled *P*. *leopoldi* reference genome with STAR [70].

Other RNA-seq data of the brain, heart, muscle, and liver from three fish species, *L*. *oculatus*, *D*. *rerio*, and *C*. *milii*, were retrieved from the NBCI Sequence Read Archive (SRA). The raw RNA-seq data were filtered using Trimmomatic 0.32 to generate clean reads [71]. Per base sequence qualities of filtered fastq files were checked with FastQC (https://www.bioinformatics.babraham.ac.uk/projects/fastqc/). The Ensembl genome of each species was used as a reference genome, and filtered reads were aligned onto the references using STAR [70].

RSEM was used to quantify expressed genes into TPM values [72]. R package DEGseq was used to detect DEGs [73]. One-to-one orthologous genes between *P*. *leopoldi* and *D*. *rerio* and web-based DAVID Bioinformatics Resources were used for GO annotation [74].

### Examination of bone formation-related gene families

The *BMP* and *BMPR* genes of zebrafish were used as query to search the BMP and BMPR gene families in *P*. *leopoldi*, *C*. *carpio*, *L*. *oculatus*, *L*. *chalumnae*, *C*. *milii*, and *B*. *floridae*. Other bone formation-related gene families were examined as follows. The zebrafish orthologous genes were used to annotate each gene family. The gene family with the keyword of “bone”, “calcium”, or “vitamin D” was kept as the bone formation-related candidate gene family for further examination. These bone formation-related candidate gene families were manually checked with literature evidence in order to find the target genes for knockdown experiment.

### *gc* gene knockdown in zebrafish

*D*. *rerio* (AB strain) was provided by an in-house *D*. *rerio* Core Facility (CAS Center for Excellence in Molecular Cell Science), and all experimental protocols were approved by the Institutional Animal Care and Use Committee. Two sgRNAs (gc-e4: 5′-GCTCAATGCCTGGATGCTTGGT-3′; gc-e8: 5′-TCGGTTTGGATTCATCGCAGGT-3′) were designed to target the sequences in the exon 4 and exon 8 of the *gc* gene in zebrafish, respectively, based on the Clustered Regularly Interspaced Short Palindromic Repeats (CRISPR) design website CCTop (https://crispr.cos.uni-heidelberg.de/index.html) and CHOPCHOP (https://chopchop.cbu.uib.no/). DNA templates for sgRNAs were produced by annealing and elongating a forward primer containing T7 promoter, guide sequence, and a reverse primer encoding the standard chimeric sgRNA scaffold [75] (Table S14). DNA templates were purified, and sgRNAs were *in vitro* synthesized and purified. Cas9 RNP complexes were prepared with Cas9 protein and sgRNAs as previously described [76]. The RNPs were injected into one-cell-stage *D*. *rerio* embryos. Each embryo was injected with a 1-nl mix containing ∼ 5 μM Cas9 and 1 μg/μl (31 μM) sgRNA.

*D*. *rerio* larvae were processed for bone staining with Alizarin Red S using a modified protocol [77]. Briefly, larvae of 6 days post-fertilization (dpf) were fixed in 4% paraformaldehyde (w/v, pH 7.4) overnight at 4 °C, washed in 1% KOH for 5 min, and bleached in 3% H_2_O_2_/0.5% KOH for 40 min. All specimens were initially stained with 0.05% Alizarin Red S in 70% ethanol overnight, and soaked thoroughly with 25% glycerol/0.1% KOH, 50% glycerol/0.1% KOH, and 75% glycerol/0.1% KOH sequentially. All specimens were stored in 80% glycerol/H_2_O.

## Data availability

The raw sequencing data reported in this study have been deposited in the Genome Sequence Archive [78] at the National Genomics Data Center (NGDC), Beijing Institute of Genomics (BIG), Chinese Academy of Sciences (CAS) / China National Center for Bioinformation (CNCB) (GSA: CRA003264) , and are publicly accessible at https://ngdc.cncb.ac.cn/gsa. The whole-genome sequence data and its annotation file in GFF format in this study have been deposited in the Genome Warehouse [79] at the NGDC, BIG, CAS / CNCB (GWH: GWHAOTN00000000), and are publicly accessible at https://ngdc.cncb.ac.cn/gwh.

## Competing interests

Xiang Zhang is the CEO of Shanghai Nanmulin Biotechnology Company Limited and pays for the sequencing of *P*. *leopoldi*. All the other authors have declared no competing interests.

## CRediT authorship contribution statement

**Jingqi Zhou:** Methodology, Formal analysis, Visualization, Funding acquisition, Writing – original draft, Writing – review & editing. **Ake Liu:** Methodology, Formal analysis, Visualization, Data curation. **Funan He:** Methodology, Formal analysis, Visualization. **Yunbin Zhang:** Formal analysis, Validation. **Libing Shen:** Conceptualization, Writing – original draft, Writing – review & editing, Supervision. **Jun Yu:** Conceptualization, Writing – review & editing, Supervision. **Xiang Zhang:** Conceptualization, Resources, Funding acquisition, Writing – review & editing, Supervision. All authors have read and approved the final manuscript.

## Supplementary material

Supplementary Table S1 **Statistics of genome sequencing data of white-blotched river stingray**

Supplementary Table S2 **Base content statistics of white-blotched river stingray genome**

Supplementary Table S3 **Characteristic statistics of genome assembly**

Supplementary Table S4 **Reads coverage statistics of white-blotched river stingray genome**

Supplementary Table S5 **Genome assembly results of white-blotched river stingray**

Supplementary Table S6 **Statistical results of repeated sequences in white-blotched river stingray genome**

Supplementary Table S7 **Statistics of repeated sequence classification in white-blotched river stingray genome**

Supplementary Table S8 **Basic statistical information for gene structure prediction**

Supplementary Table S9 **The gene function annotation using different methods**

Supplementary Table S10 **Gene structures in eleven fish species**

Supplementary Table S11 **Statistics of non-coding RNAs in white-blotched river stingray genome**

Supplementary Table S12 **Evolutionary rate evaluated by Tajima’s rate tests among 26 species**

Supplementary Table S13 **The number of BMPs and BMP receptors in seven species**

Supplementary Table S14 **Primer sequences used to generate sgRNAs used in this study**
