# Supplementary material for: Draft Genome of White-blotched River Stingray Provides Novel Clues for Niche Adaptation and Skeleton Formation
Source: Genomics Proteomics Bioinformatics. 2022 Dec 5;21(3):501–14. doi: 10.1016/j.gpb.2022.11.005 (PMC10787021; doi:10.1016/j.gpb.2022.11.005)
Supplement: Supplementary Table S1 — Statistics of genome sequencing data of white-blotched river stingray [file mmc1.docx]

**Table S1 Statistics of genome sequencing data of white-blotched river stingray**

| **Paired-end libraries** | **Insert size** | **Total data (Gb)** | **Read length (bp)** | **Sequence coverage (×)** |
| --- | --- | --- | --- | --- |
| Illumina reads | 250 bp | 293.12 |  | 66.77 |
|  | 350 bp | 288.66 | 150 | 65.75 |
|  | 450 bp | 300.41 |  | 68.43 |
| PacBio reads | 20 kb | 270.51 | － | 61.61 |
| 10X Genomics |  | 475.93 | 150 | 108.41 |
| Total | － | 1628.63 | － | 370.97 |
