# Supplementary material for: Draft Genome of White-blotched River Stingray Provides Novel Clues for Niche Adaptation and Skeleton Formation
Source: Genomics Proteomics Bioinformatics. 2022 Dec 5;21(3):501–14. doi: 10.1016/j.gpb.2022.11.005 (PMC10787021; doi:10.1016/j.gpb.2022.11.005)
Supplement: Supplementary Table S2 — Base content statistics of white-blotched river stingray genome [file mmc2.docx]

**Table S2**  **Base content statistics of white-blotched river stingray genome**

|  | **Number (bp)** | **% of genome** |
| --- | --- | --- |
| A | 1,253,750,658 | 28.77 |
| T | 1,254,374,523 | 28.79 |
| C | 914,070,220 | 20.98 |
| G | 914,572,508 | 20.99 |
| N | 20,623,492 | 0.47 |
| Total | 4,357,391,401 |  |
| GC | 1,828,642,728 | 41.97 |

*Note*: GC content of the genome without N.
