# Supplementary material for: Draft Genome of White-blotched River Stingray Provides Novel Clues for Niche Adaptation and Skeleton Formation
Source: Genomics Proteomics Bioinformatics. 2022 Dec 5;21(3):501–14. doi: 10.1016/j.gpb.2022.11.005 (PMC10787021; doi:10.1016/j.gpb.2022.11.005)
Supplement: Supplementary Table S3 — Characteristic statistics of genome assembly [file mmc3.docx]

**Table S3 Characteristic statistics of genome assembly**

| **Item** | **Feature** |
| --- | --- |
| Kmer | 17 |
| Depth | 29 |
| Genome size (Mb) | 4231.24 |
| Revised genome size (Mb) | 4209.61 (4.11 Gb) |
| Heterozygous rate (%) | 0.79 |
| Repeat rate (%) | 73.87 |
| Q30 | 99.9 |

*Note*: Q30, The percentage of bases in cleaned reads with quality score 30 or greater than 30.
