# Supplementary material for: Draft Genome of White-blotched River Stingray Provides Novel Clues for Niche Adaptation and Skeleton Formation
Source: Genomics Proteomics Bioinformatics. 2022 Dec 5;21(3):501–14. doi: 10.1016/j.gpb.2022.11.005 (PMC10787021; doi:10.1016/j.gpb.2022.11.005)
Supplement: Supplementary Table S4 — Reads coverage statistics of white-blotched river stingray genome [file mmc4.docx]

**Table S4**  **Reads coverage statistics of white-blotched river stingray genome**

|  |  | **Percentage** |
| --- | --- | --- |
| Reads | Mapping rate (%) | 98.48 |
| Genome | Average sequencing depth | 50.91 |
|  | Coverage (%) | 98.74 |
|  | Coverage at least 4× (%) | 97.84 |
|  | Coverage at least 10× (%) | 95.72 |
|  | Coverage at least 20× (%) | 89.03 |

*Note*: Mapping rate means the percentage of reads mapping to genome. Average sequence depth means the average sequence depth of each base mapped by reads in genome. Coverage means the percentage of genome coverage mapped by reads. Coverage at least N× means the percentage of genome coverage mapped by N× reads.
