# Supplementary material for: Draft Genome of White-blotched River Stingray Provides Novel Clues for Niche Adaptation and Skeleton Formation
Source: Genomics Proteomics Bioinformatics. 2022 Dec 5;21(3):501–14. doi: 10.1016/j.gpb.2022.11.005 (PMC10787021; doi:10.1016/j.gpb.2022.11.005)
Supplement: Supplementary Table S5 — Genome assembly results of white-blotched river stingray [file mmc5.docx]

**Table S5**  **Genome assembly results of white-blotched river stingray**

| **Sample ID** | **Length** | | **Number** | |
| --- | --- | --- | --- | --- |
|  | **Contig (bp)** | **Scaffold (bp)** | **Contig** | **Scaffold** |
| Total | 4,336,767,909 | 4,357,391,401 | 16,227 | 13,238 |
| Max | 34,999,168 | 41,069,189 | － | － |
| Number >= 2000 | － | － | 15,784 | 12,796 |
| N50 | 3,937,865 | 5,675,171 | 222 | 179 |
| N60 | 2,226,993 | 3,630,306 | 367 | 275 |
| N70 | 1,057,949 | 1,818,790 | 649 | 446 |
| N80 | 359,267 | 769,167 | 1366 | 814 |
| N90 | 113,796 | 191,036 | 3662 | 1982 |
