# Supplementary material for: Draft Genome of White-blotched River Stingray Provides Novel Clues for Niche Adaptation and Skeleton Formation
Source: Genomics Proteomics Bioinformatics. 2022 Dec 5;21(3):501–14. doi: 10.1016/j.gpb.2022.11.005 (PMC10787021; doi:10.1016/j.gpb.2022.11.005)
Supplement: Supplementary Table S6 — Statistical results of repeated sequences in white-blotched river stingray genome [file mmc6.docx]

**Table S6**  **Statistical results of repeated sequences in white-blotched river stingray genome**

| **Type** | **Repeat size (bp)** | **% of genome** |
| --- | --- | --- |
| Tandem Repeats Finder | 186,908,778 | 4.29 |
| RepeatMasker | 2,998,926,446 | 68.82 |
| ProteinMask | 62,564,836 | 1.44 |
| Total | 3,119,184,614 | 71.58 |

*Note*: Total repeated sequences are the results obtained by the above methods, which are the non-redundant result after removing the overlapped parts among three methods.
