# Supplementary material for: Draft Genome of White-blotched River Stingray Provides Novel Clues for Niche Adaptation and Skeleton Formation
Source: Genomics Proteomics Bioinformatics. 2022 Dec 5;21(3):501–14. doi: 10.1016/j.gpb.2022.11.005 (PMC10787021; doi:10.1016/j.gpb.2022.11.005)
Supplement: Supplementary Table S7 — Statistics of repeated sequence classification in white-blotched river stingray genome [file mmc7.docx]

**Table S7**  **Statistics of repeated sequence classification in white-blotched river stingray genome**

|  | ***Denovo* + Repbase length (bp)** | **% in Genome** | **TE proteins length (bp)** | **% in Genome** | **Combined TEs length (bp)** | **% in Genome** |
| --- | --- | --- | --- | --- | --- | --- |
| DNA | 19,792,076 | 0.45 | 22,913,194 | 0.53 | 42,705,270 | 0.98 |
| LINE | 2,189,913,381 | 50.26 | 61,603,347 | 1.41 | 2,251,516,728 | 51.67 |
| SINE | 721,756 | 0.02 | 0 | 0 | 721,756 | 0.02 |
| LTR | 1,064,263,616 | 24.42 | 32,380,894 | 0.74 | 1,096,644,510 | 25.16 |
| Simple repeat | 29,991,064 | 0.69 | 0 | 0 | 29,991,064 | 0.69 |
| Unknown | 4,264,845 | 0.10 | 0 | 0 | 4,264,845 | 0.10 |
| Total | 2,998,926,446 | 68.82 | 62,564,836 | 1.44 | 3,061,491,282 | 70.26 |

*Note*: *Denovo* + Repbase are the transposon elements obtained by annotating the genome using RepeatMasker software after integrating the library predicted by RepeatModeler, RepeatScout, and LTR_FINDER combined with the Repbase nucleotide database using Uclust software according to the 80-80-80 principle. TE proteins are the transposon elements obtained by annotating the genome using RepeatProteinMask software based on the Repbase protein library. Combined TEs are the results of integrating the above two methods and removing redundancy. Unknown means that the repeat sequence cannot be classified by RepeatMasker. SINE, short interspersed element; LINE, long interspersed element; LTR, long terminal repeat; TE, transposon element.
