# Supplementary material for: Draft Genome of White-blotched River Stingray Provides Novel Clues for Niche Adaptation and Skeleton Formation
Source: Genomics Proteomics Bioinformatics. 2022 Dec 5;21(3):501–14. doi: 10.1016/j.gpb.2022.11.005 (PMC10787021; doi:10.1016/j.gpb.2022.11.005)
Supplement: Supplementary Table S8 — Basic statistical information for gene structure prediction [file mmc8.docx]

**Table S8**  **Basic statistical information for gene structure prediction**

|  | **Gene set** | **Number** | **Average transcript length (bp)** | **Average CDS length (bp)** | **Average exons per gene** | **Average exon length (bp)** | **Average intron length (bp)** |
| --- | --- | --- | --- | --- | --- | --- | --- |
| *De novo* | AUGUSTUS | 56,209 | 32,106.89 | 910.51 | 3.65 | 249.50 | 11,774.89 |
|  | GlimmerHMM | 406,617 | 9631.17 | 376.45 | 2.68 | 140.42 | 5506.02 |
|  | SNAP | 105,143 | 48,727.62 | 574.33 | 3.35 | 171.65 | 20,526.24 |
|  | Geneid | 66,828 | 11,027.78 | 495.14 | 3.13 | 158.19 | 4944.57 |
|  | GENSCAN | 67,768 | 41,011.58 | 1118.54 | 5.39 | 207.34 | 9077.23 |
| Homolog | *Branchiostoma floridae* | 155,108 | 2183.17 | 398.93 | 1.36 | 293.92 | 4993.95 |
|  | *Callorhinchus milii* | 66,757 | 12,458.32 | 851.55 | 2.91 | 292.66 | 6077.74 |
|  | *Danio rerio* | 47,991 | 10,643.20 | 978.27 | 2.92 | 334.45 | 5020.84 |
|  | *Gadus morhua* | 36,147 | 15,776.59 | 861.76 | 3.46 | 249.02 | 6061.55 |
|  | *Gasterosteus aculeatus* | 51,120 | 11,872.87 | 696.79 | 2.90 | 240.63 | 5895.56 |
|  | *Latimeria chalumnae* | 76,153 | 7199.62 | 924.28 | 2.21 | 417.76 | 5175.57 |
|  | *Oreochromis niloticus* | 108,253 | 5871.07 | 855.05 | 2.07 | 413.42 | 4695.47 |
|  | *Oryzias latipes* | 105,441 | 6252.47 | 838.19 | 1.98 | 423.47 | 5528.52 |
|  | *Takifugu rubripes* | 82,069 | 9685.26 | 870.88 | 2.36 | 369.65 | 6500.54 |
|  | *Tetraodon nigroviridis* | 27,261 | 20,922.13 | 1099.54 | 4.32 | 254.45 | 5968.32 |
|  | PASA | 81,109 | 50,355.53 | 1186.46 | 6.99 | 169.64 | 8202.94 |
| RNA sequencing | Cufflinks | 70,035 | 67,076.77 | 3854.73 | 8.19 | 470.43 | 8788.14 |
|  | 66,210 | 21,378.76 | 814.38 | 3.55 | 229.11 | 8049.99 |  |
| EVidenceModeler | | 64,180 | 25,776.76 | 859.97 | 3.81 | 225.42 | 8851.47 |
| Pasa-update | | 23,240 | 57,255.91 | 1343.22 | 7.63 | 176.02 | 8431.71 |

*Note*: It contains UTR region but no other regions. It obtains from the result of Pasa-update after removing the variable isoforms to keep the longest transcript and removing the redundant single exon. The filtering conditions: TE overlap no less than 20%, early termination, supported only by *de novo* evidence, and RPKM expression less than 1 in each tissue. CDS, coding sequence; UTR, untranslated region; RPKM, reads per kilobase per million mapped reads.
