# Supplementary material for: Draft Genome of White-blotched River Stingray Provides Novel Clues for Niche Adaptation and Skeleton Formation
Source: Genomics Proteomics Bioinformatics. 2022 Dec 5;21(3):501–14. doi: 10.1016/j.gpb.2022.11.005 (PMC10787021; doi:10.1016/j.gpb.2022.11.005)
Supplement: Supplementary Table S9 — The gene function annotation using different methods [file mmc9.docx]

**Table S9 The gene function annotation uses different methods**

|  | **Number** | **Percent (%)** |
| --- | --- | --- |
| Total | 23,240 | - |
| Swiss-Prot | 19,440 | 83.60 |
| NR | 21,190 | 91.20 |
| KEGG | 18,385 | 79.10 |
| InterPro | 22,900 | 98.50 |
| GO | 21,040 | 90.50 |
| Pfam | 16,883 | 72.60 |
| Annotated | 23,030 | 99.10 |
| Unannotated | 210 | 0.90 |

*Note*: NR, Non-Redundant Protein Sequence Database; KEGG, Kyoto Encyclopedia of Genes and Genomes; GO, Gene Ontology
