# Supplementary material for: Draft Genome of White-blotched River Stingray Provides Novel Clues for Niche Adaptation and Skeleton Formation
Source: Genomics Proteomics Bioinformatics. 2022 Dec 5;21(3):501–14. doi: 10.1016/j.gpb.2022.11.005 (PMC10787021; doi:10.1016/j.gpb.2022.11.005)
Supplement: Supplementary Table S10 — Gene structures in eleven fish species [file mmc10.docx]

**Table S10**  **Gene structures in eleven fish species**

| **Species** | **Number** | **Average transcript length (bp)** | **Average CDS length (bp)** | **Average exons per gene** | **Average exon length (bp)** | **Average intron length (bp)** |
| --- | --- | --- | --- | --- | --- | --- |
| Bfl | 28,621 | 9236.65 | 1392.24 | 7.03 | 198.11 | 1301.41 |
| Cmi | 17,847 | 24,214.70 | 1668.28 | 9.87 | 169.06 | 2542.48 |
| Dre | 25,619 | 25,207.59 | 1642.64 | 9.42 | 174.39 | 2798.97 |
| Gac | 20,787 | 8451.06 | 1548.67 | 10.4 | 148.94 | 734.44 |
| Gmo | 20,095 | 15,245.21 | 1459.03 | 12.72 | 114.67 | 1175.90 |
| Lch | 19,569 | 36,970.26 | 1562.50 | 9.95 | 157.11 | 3958.36 |
| Ola | 19,699 | 12,145.58 | 1515.82 | 10.25 | 147.82 | 1148.61 |
| Oni | 21,437 | 14,903.11 | 1714.22 | 10.9 | 157.25 | 1332.07 |
| Ple | 23,240 | 57,255.91 | 1343.22 | 7.63 | 176.02 | 8431.71 |
| Tni | 19,602 | 6066.17 | 1516.59 | 10.52 | 144.2 | 478.02 |
| Tru | 18,523 | 7492.75 | 1693.53 | 11.1 | 152.61 | 574.33 |

*Note*: Bfl, *Branchiostoma floridae*; Cmi, *Callorhinchus milii*; Dre, *Danio rerio*; Gac, *Gasterosteus aculeatus*; Gmo, *Gadus morhua*; Lch, *Latimeria chalumnae*; Ola, *Oryzias latipes*; Oni, *Oreochromis niloticus*; Ple, *Potamotrygon Leopoldi*; Tni, *Tetraodon nigroviridis*; Tru, *Takifugu rubripes*.
