# Supplementary material for: Draft Genome of White-blotched River Stingray Provides Novel Clues for Niche Adaptation and Skeleton Formation
Source: Genomics Proteomics Bioinformatics. 2022 Dec 5;21(3):501–14. doi: 10.1016/j.gpb.2022.11.005 (PMC10787021; doi:10.1016/j.gpb.2022.11.005)
Supplement: Supplementary Table S11 — Statistics of non-coding RNAs in white-blotched river stingray genome [file mmc11.docx]

**Table S11**  **Statistics of non-coding RNAs in white-blotched river stingray genome**

|  | **Type** | **Copy** | **Average length (bp)** | **Total length (bp)** | **% of genome** |
| --- | --- | --- | --- | --- | --- |
| miRNA | | 1262 | 101.18 | 127,691 | 0.002930 |
| tRNA | | 2729 | 75.87 | 207,038 | 0.004751 |
| rRNA | rRNA | 2405 | 176.59 | 424,687 | 0.009746 |
|  | 18S | 712 | 196.25 | 139,731 | 0.003207 |
|  | 28S | 1653 | 170.08 | 281,136 | 0.006452 |
|  | 5.8S | 9 | 130 | 1170 | 0.000027 |
|  | 5S | 31 | 85.48 | 2650 | 0.000061 |
| snRNA | snRNA | 871 | 107.66 | 93,775 | 0.002152 |
|  | CD-box | 84 | 100.83 | 8470 | 0.000194 |
|  | HACA-box | 83 | 172.92 | 14,352 | 0.000329 |
|  | splicing | 690 | 99.30 | 68,514 | 0.001572 |

*Note*: miRNA, microRNAs; tRNA, transfer RNA; rRNA, ribosomal RNA; snRNA, small nuclearRNA.
