# Supplementary material for: Draft Genome of White-blotched River Stingray Provides Novel Clues for Niche Adaptation and Skeleton Formation
Source: Genomics Proteomics Bioinformatics. 2022 Dec 5;21(3):501–14. doi: 10.1016/j.gpb.2022.11.005 (PMC10787021; doi:10.1016/j.gpb.2022.11.005)
Supplement: Supplementary Table S12 — Evolutionary rate evaluated by Tajima’s rate tests among 26 species [file mmc12.docx]

**Table S12 Evolutionary rate evaluated by Tajima’s rate tests among 26 species**

| Outgroup | Group A | Group B | Identical sites | Divergent sites | Unique difference in outgroup | Unique difference in A | Unique difference in B | Chi-square test statistics | *P* value | Conclusion |
| --- | --- | --- | --- | --- | --- | --- | --- | --- | --- | --- |
| BRF | PMA | PLE | 27,261 | 6184 | 7123 | 4341 | 3293 | 143.87 | 0 | PLE slower |
| BRF | CMI | PLE | 29,958 | 3363 | 11,652 | 1644 | 1585 | 1.08 | 0.299 | No difference |
| BRF | LAC | PLE | 29,050 | 4413 | 9920 | 2552 | 2267 | 16.86 | 0.00004 | PLE slower |
| BRF | LOC | PLE | 29,458 | 4313 | 9940 | 2144 | 2347 | 9.18 | 0.00245 | LOC slower |
| BRF | DAR | PLE | 29,002 | 4614 | 9517 | 2600 | 2469 | 3.39 | 0.065 | No difference |
| PMA | CMI | PLE | 32,538 | 2976 | 9072 | 1846 | 1770 | 1.6 | 0.206 | No difference |
| PMA | LAC | PLE | 31,522 | 3880 | 7448 | 2862 | 2490 | 25.86 | 0 | PLE slower |
| PMA | LOC | PLE | 31,965 | 3664 | 7433 | 2419 | 2721 | 17.74 | 0.00003 | LOC slower |
| PMA | DAR | PLE | 31,464 | 4026 | 7055 | 2920 | 2737 | 5.92 | 0.015 | PLE slower |
| PMA | BRF | PLE | 27,261 | 6184 | 4341 | 7123 | 3293 | 1409.3 | 0 | PLE slower |

*Note*: BRF, amphioxus; PMA, sea lamprey; CMI, elephant shark; LAC, coelacanth; LOC, spotted gar; DAR, zebrafish; PLE, white-blotched river stingray.
