# Supplementary material for: Draft Genome of White-blotched River Stingray Provides Novel Clues for Niche Adaptation and Skeleton Formation
Source: Genomics Proteomics Bioinformatics. 2022 Dec 5;21(3):501–14. doi: 10.1016/j.gpb.2022.11.005 (PMC10787021; doi:10.1016/j.gpb.2022.11.005)
Supplement: Supplementary Table S13 — The number of BMPs and BMP receptors in seven species [file mmc13.docx]

**Table S13 The number of BMPs and BMP receptors in seven species**

|  | BRF | CMI | PLE | LAC | LOC | CCA | DAR |
| --- | --- | --- | --- | --- | --- | --- | --- |
| ENSDARG00000103679 bone morphogenetic protein 16 [Source:ZFIN;Acc:ZDB-GENE-100115-1] | 0 | 0 | 0 | 1 | 1 | 3 | 1 |
| ENSDARG00000060526 bone morphogenetic protein 3 [Source:ZFIN;Acc:ZDB-GENE-030131-7192] ENSDARG00000109238 bone morphogenetic protein 3 [Source:ZFIN;Acc:ZDB-GENE-030131-7192] | 0 | 1 | 1 | 1 | 1 | 2 | 2 |
| ENSDARG00000013409 bone morphogenetic protein 2a [Source:ZFIN;Acc:ZDB-GENE-980526-388] ENSDARG00000019995 bone morphogenetic protein 4 [Source:ZFIN;Acc:ZDB-GENE-980528-2059] ENSDARG00000041430 bone morphogenetic protein 2b [Source:ZFIN;Acc:ZDB-GENE-980526-474] ENSDARG00000115191 bone morphogenetic protein 2b [Source:ZFIN;Acc:ZDB-GENE-980526-474] | 1 | 4 | 2 | 2 | 2 | 5 | 4 |
| ENSDARG00000037491 bone morphogenetic protein 15 [Source:ZFIN;Acc:ZDB-GENE-030131-6115] | 0 | 1 | 0 | 1 | 1 | 2 | 1 |
| ENSDARG00000028053 bone morphogenetic protein 1b [Source:ZFIN;Acc:ZDB-GENE-060818-2] ENSDARG00000028071 bone morphogenetic protein 1a | 2 | 4 | 4 | 3 | 3 | 3 | 4 |
| ENSDARG00000061769 bone morphogenetic protein 10 [Source:ZFIN;Acc:ZDB-GENE-060526-211] | 0 | 2 | 1 | 1 | 1 | 1 | 1 |
| ENSDARG00000015686 bone morphogenetic protein 6 [Source:ZFIN;Acc:ZDB-GENE-050306-42] ENSDARG00000018260 bone morphogenetic protein 7a [Source:ZFIN;Acc:ZDB-GENE-000208-25] ENSDARG00000035677 bone morphogenetic protein 8a [Source:ZFIN;Acc:ZDB-GENE-030912-13] ENSDARG00000063230 bone morphogenetic protein 7b [Source:ZFIN;Acc:ZDB-GENE-060929-328] ENSDARG00000101701 bone morphogenetic protein 5 [Source:ZFIN;Acc:ZDB-GENE-040426-1413] | 1 | 4 | 4 | 3 | 4 | 12 | 5 |
| ENSDARG00000044924 growth differentiation factor 11 [Source:ZFIN;Acc:ZDB-GENE-040427-2] bmp11 | 1 | 2 | 2 | 2 | 2 | 6 | 4 |
| ENSDARG00000002760 growth differentiation factor 5 [Source:ZFIN;Acc:ZDB-GENE-990415-39] bmp14 ENSDARG00000005510 growth differentiation factor 6b [Source:ZFIN;Acc:ZDB-GENE-980526-442] bmp13b ENSDARG00000042784 growth differentiation factor 7 [Source:ZFIN;Acc:ZDB-GENE-990714-1] bmp12 ENSDARG00000053479 growth differentiation factor 6a [Source:ZFIN;Acc:ZDB-GENE-980526-373] bmp13a | 1 | 3 | 3 | 3 | 3 | 13 | 4 |
| ENSDARG00000059173 growth differentiation factor 2 [Source:ZFIN;Acc:ZDB-GENE-100107-1] BMP9 | 0 | 1 | 1 | 1 | 1 | 3 | 1 |
| Total | 6 | 22 | 18 | 18 | 19 | 50 | 27 |

*Note*: BMP, bone morphogenetic protein; CCA, common carp.
