# Supplementary material for: Draft Genome of White-blotched River Stingray Provides Novel Clues for Niche Adaptation and Skeleton Formation
Source: Genomics Proteomics Bioinformatics. 2022 Dec 5;21(3):501–14. doi: 10.1016/j.gpb.2022.11.005 (PMC10787021; doi:10.1016/j.gpb.2022.11.005)
Supplement: Supplementary Table S14 — Primer sequences used to generate sgRNAs used in this study [file mmc14.docx]

**Table S14**  **Primer sequences used to generate sgRNAs used in this study**

|  | **Sequence (5'→ 3')** |
| --- | --- |
| sgRNA scaffold primer | AAAAGCACCGACTCGGTGCCACTTTTTCAAGTTGATAACGGACTAGCCTTATTTTAACTTGCTATTTCTAGCTCTAAAAC |
| Experimental guide  template primers |  |
| Control (EGFP) | TAATACGACTCACTATAGGCGAGGGCGATGCCACCTAGTTTTAGAGCTAGAAATAGC |
| gc-e4 | TAATACGACTCACTATAGGCTCAATGCCTGGATGCTTGGTTTTAGAGCTAGAAATAGC |
| gc-e8 | TAATACGACTCACTATAGGTCGGTTTGGATTCATCGCAGGTTTTAGAGCTAGAAATAGC |

*Note*: sgRNA target sequences are underlined. EGFP, enhanced green fluorescent protein.
